# UV-Vis Spectroscopy, Electrochemical and DFT Study of Tris(β-diketonato)iron(III) Complexes with Application in DSSC: Role of Aromatic Thienyl Groups

**DOI:** 10.3390/molecules27123743

**Published:** 2022-06-10

**Authors:** Marrigje M. Conradie

**Affiliations:** Department of Chemistry, University of the Free State, P.O. Box 339, Bloemfontein 9300, South Africa; conradiemm@ufs.ac.za

**Keywords:** iron(III), UV-Vis, DFT, spectroelectrochemistry, acetylacetone, dye-sensitized solar cells

## Abstract

A series of tris(β-diketonato)iron(III) complexes, with the β-diketonato ligand bearing different substituent groups, have been synthesized and characterized by Fourier transform infrared (FT-IR), ultraviolet-visible (UV-Vis) and mass spectroscopic methods. The maximum band UV-Vis absorption wavelengths of the tris(β-diketonato)iron(III) complexes were in the range of 270–380 nm. The complexes have very good solubility in various solvents such as chloroform, dichloromethane, ethyl acetate, tetrahydrofurane, dimethylsulphoxide and dimethylformamide. After the syntheses and characterization processes, spectroscopic and electrochemical properties of these tris(β-diketonato)iron(III) complexes were investigated. A density functional theory (DFT) study related to the spectroscopic and electrochemical properties of the tris(β-diketonato)iron(III) complexes was used to investigate the possible application of these complexes as dye sensitizers or redox mediators in dye-sensitized solar cells.

## 1. Introduction

Tris(β-diketonato)iron(III) complexes represent an important class of organometallic compounds, which have been used in modern applications by many researchers in recent years. Some of the modern application areas where these compounds are used include photocatalysis [1], redox mediators in solar cells [2], energy storage (batteries) [3,4,5], catalysts, pre-catalysts and reagents in organic chemistry [6,7,8,9].

Iron(II) and iron(III) complexes have strong absorption bands in the near ultraviolet region. Iron porphyrins, for example, have a typical Soret band at ca. 400 nm [10], whereas tris(β-diketonato)iron(III) complexes have a strong absorption band at ca. 300 nm [11,12]. The intensity and position of the bands change as the iron complexes are reduced or oxidized [10,13,14,15]. Tris(β-diketonato)iron(III) complexes undergo a single one-electron iron-related electrochemically and chemically reversible reduction reaction. The electrochemical data of a series of tris(β-diketonato)iron(III) complexes were previously reported [16]. To compliment the electrochemical study of the tris(β-diketonato)iron(III) complexes, a spectroscopic and spectroelectrochemical (SEC) study was conducted on the complexes. SEC combines electrochemistry and spectroscopy to show the influence of the redox chemistry of the tris(β-diketonato)iron(III) complexes on their UV-Vis absorption spectra. The oxidation state of the iron complexes is changed electrochemically, while the spectra of the product of the redox transformation are then simultaneously monitored in situ by UV-Vis spectroscopy.

**Scheme 1:**
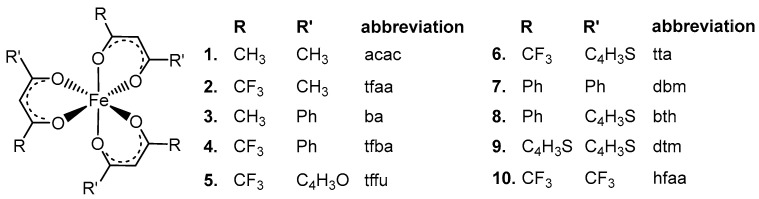
[Fe^III^(β-diketonato)_3_] complexes (**1**)–(**10**) studied, where β-diketonato = (RCOCHCOR’)^−^. For complexes containing unsymmetrical β-diketonato ligands (**2**–**6** and **8**), two isomers (*fac* and *mer*) are possible. The structure of the *fac*-isomer is shown for complexes **2**–**6** and **8**.

In this study, we thus present a spectroscopic, electrochemical and computational chemistry study of the series of tris(β-diketonato)iron(III) complexes shown in Scheme 1. The influence of the type of substituent groups (methyl, trifluoromethyl, furyl, phenyl and thienyl) on the observed spectroscopic and electrochemical behaviour is evaluated. Redox and spectral properties are evaluated for possible application in dye-sensitized solar cells (DSSC).

## 2. Results and Discussion

The molecular structure of complexes **2**–**6** and **8**, containing β-diketonato ligands with two different substituent groups, can be a *fac*- or a *mer*-isomer [17,18]. The difference in the density functional theory (DFT) calculated electronic energies (E), zero-point corrected electronic energies (ZEE) and free energies (G) of the *fac*- and *mer*-isomers of a complex are generally very small (Table 1), implying that both isomers will exist in an experimental sample of a complex. The experimentally measured UV-Vis and redox potential are thus expected to be the result of a mixture of *fac*- and *mer*-isomers.

### 2.1. UV-Vis Spectroscopy

#### 2.1.1. Experimental Spectra

**Figure 1:**
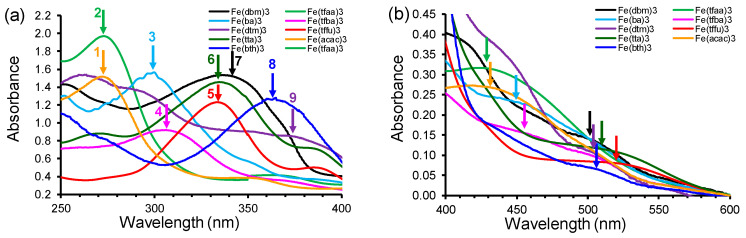
Experimental UV-Vis of 1–9 in (**a**) the 250–400 nm and (**b**) the 400–600 nm region.

The experimental spectra of complexes **1**–**9** have a strong absorbance peak in the range of 270–376 nm, with one or more lower energy weak absorbance bands in the 400–600 nm region (see Figure 1). Comparing the UV-Vis of complexes **1**–**9**, it is clear that λ_A,max,exp_ becomes red-shifted as more aromatic groups are attached to the β-diketonato ligands in the [Fe(β-diketonato)_3_] complexes **1**–**9**. The introduction of one Ph or two Ph side groups per β-diketonato ligand successively causes a redshift of ~30 nm:

[Fe(acac)_3_] (1) (270 nm) → [Fe(ba)_3_] (3) (298 nm) → [Fe(dbm)_3_] (7) (336 nm)
[Fe(tfaa)_3_] (2) (271 nm) → [Fe(tfba)_3_] (4) (304 nm) → [Fe(dbm)_3_] (7) (336 nm)

Similarly, the introduction of one or two thienyl side groups per β-diketonato ligand successively causes a redshift of more than 40 nm:

[Fe(hfaa)_3_] (10) (278 nm, calc) → [Fe(tta)_3_] (6) (333 nm) → [Fe(dtm)_3_] (9) (376 nm)

The absorbance maxima of the complex with six thienyl groups is the most red-shifted.

Complexes 1–9 can thus be grouped into three groups according to the influence of aromatic groups on the wavelength of the maximum absorbance λ_A,max,exp_, in the 250–400 nm region:

UV-Vis group 1: λ_A,max,exp_ ≈ 270 nm, complexes **1** and **2**, containing only CF_3_ or CH_3_ (no aromatic) substituent groups on the β-diketonato ligands.

UV-Vis group 2: λ_A,max,exp_ = 298–333 nm, complexes **3**–**6**, containing one aromatic substituent group (phenyl, thienyl or furyl) on each β-diketonato ligand.

UV-Vis group 3: λ_A,max,exp_ = 336–376 nm, complexes **7**–**9**, containing two aromatic substituent groups (phenyl or thienyl) on each β-diketonato ligand.

#### 2.1.2. TDDFT

**Figure 2:**
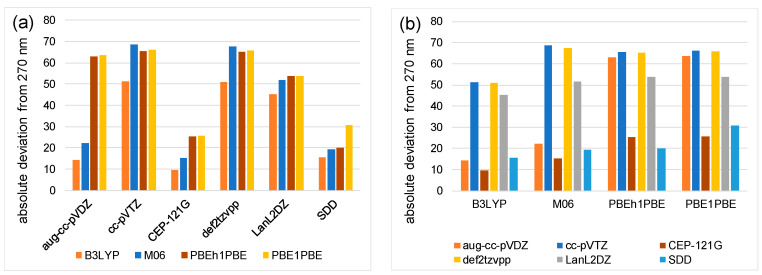
Absolute difference between the DFT-calculated maximum wavelength (λ_A,max_(calc) in nm) and the experimental value λ_A,max_(experimental) = 270 nm of [Fe(acac)_3_], complex **1**, using the indicated DFT functional and basis set combinations. Graph in (**a**) illustrates the influence of the basis set, and in (**b**) the influence of the functional, on the absolute deviation of λ_max_(calc) from λ_max_(experimental).

To get insight in the type of charge transfer (CT) bands observed in the experimental UV-Vis spectra of 1–10, a time-dependent density functional theory (TDDFT) study was performed. To validate the TDDFT method, different functionals and basis sets were used to determine the artificial spectra, excitation energies and oscillator strengths associated with the different absorbance bands for [Fe(acac)_3_], complex **1**. All excitations above 200 nm were determined. The wavelength (λ_A,max_) corresponding to the maximum intensity (oscillator strength f) and the corresponding f data are provided in Table S1. The difference between the DFT-calculated maximum wavelength (λ_A,max_(calc) in nm) and the experimental value λ_A,max_(experimental) = 270 nm of [Fe(acac)_3_], complex **1**, using a selection of functionals and basis sets that have been previously proven to give good agreement between theory and experiment [19], is shown in Figure 2. The B3LYP and M06 functionals, both using the CEP-121G basis set, gave the best agreement with the experiment, namely within 9.7 and 15.1 nm of the experimental wavelengths, respectively. Adding Grimme’s dispersion D3 to B3LYP using the CEP-121G basis set resulted in λ_A,max_(calc) = 257.7 nm for 1, with a larger deviation from the experiment (12.3 nm) than using B3LYP without D3.

**Figure 3:**
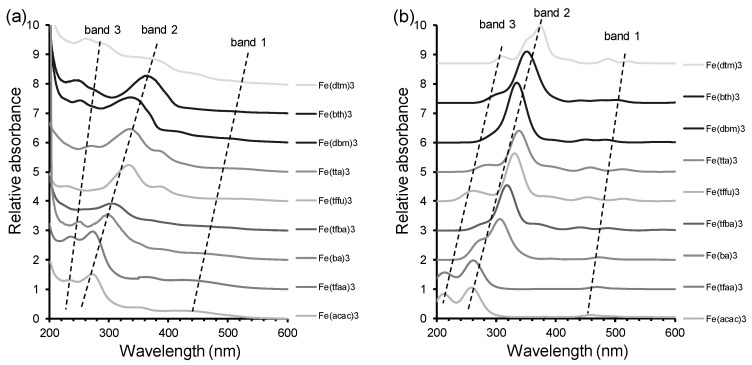
(**a**) Experimental and (**b**) B3LYP/CEP-121G-calculated time-dependent density functional theory (TDDFT) UV-Vis spectra in acetonitrile of complexes **1**–**9**. The spectra of the *mer*-isomers of 2–6 and 8 are shown in (**b**). Calculated spectra in (**b**) were generated using Gaussian broadening with a bandwidth at half maximum of 30 nm.

The results for λ_A,max_(calculated), using the B3LYP/CEP-121G and M06/CEP-121G methods for complexes **1**–**10**, are summarized in Table 1. Results that illustrate the influence of a long-range corrected functional, using CAM-B3LYP/CEP-121G, are also included in Table 1. B3LYP reproduced the λ_A,max_(experimental) for 1–9 with an average deviation (AD) and a mean absolute deviation (MAD) from experiment of 7.8 and 3.8 Å, while M06 was slightly less accurate with AD = 11.0 Å and MAD = 4.3 Å. λ_A,max_ values determined with CAM-B3LYP/CEP-121G were blue-shifted compared to the experimental values, presenting much larger values of AD = 18.4 Å and MAD = 11.6 Å. Furthermore, it is observed that, in most cases, λ_A,max_(calc) of the *fac*- and *mer*-isomers of a complex are very similar. The experimental and B3LYP-calculated spectra of 1–9 are compared in Figure 3. Both experimental and calculated spectra show three main bands (indicated as bands 1–3 in Figure 3), namely the low energy band in the visible region, the strong absorbance peak and another higher energy (smaller wavelength) band in the UV region.

**Figure 4:**
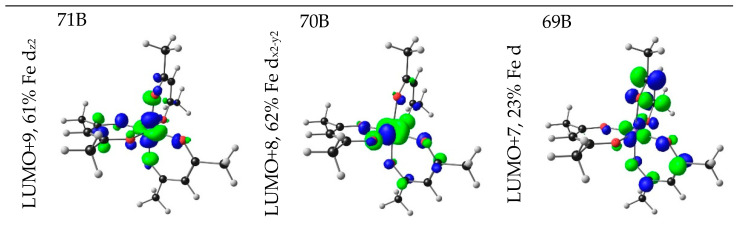

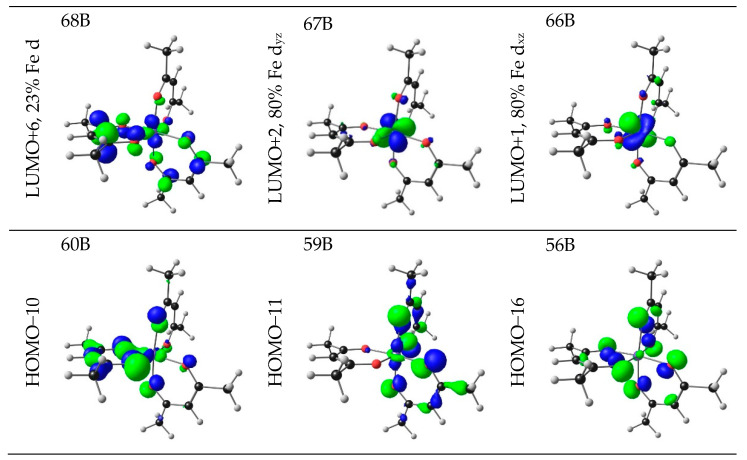
Selected Kohn−Sham molecular orbitals (MOs) of the B3LYP/CEP-121G optimized molecule 1. A contour of 0.06 Åe^−^^3^ was used for the MO plots. Color scheme: Fe (purple), O (red), C (black) and H (white).

**Figure 5:**
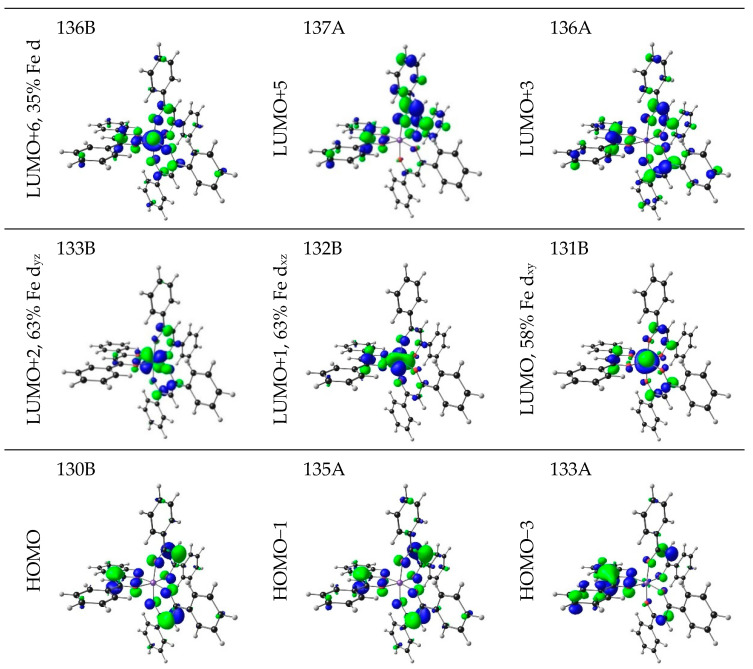

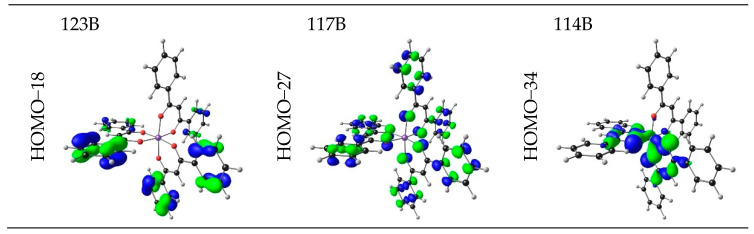
Selected Kohn−Sham molecular orbitals (MOs) of the B3LYP/CEP-121G optimized molecule 7. A contour of 0.04 Åe^−^^3^ was used for the MO plots. Color scheme used for atoms (online version): Fe (purple), O (red), C (black), S (yellow) and H (white).

**Figure 6:**
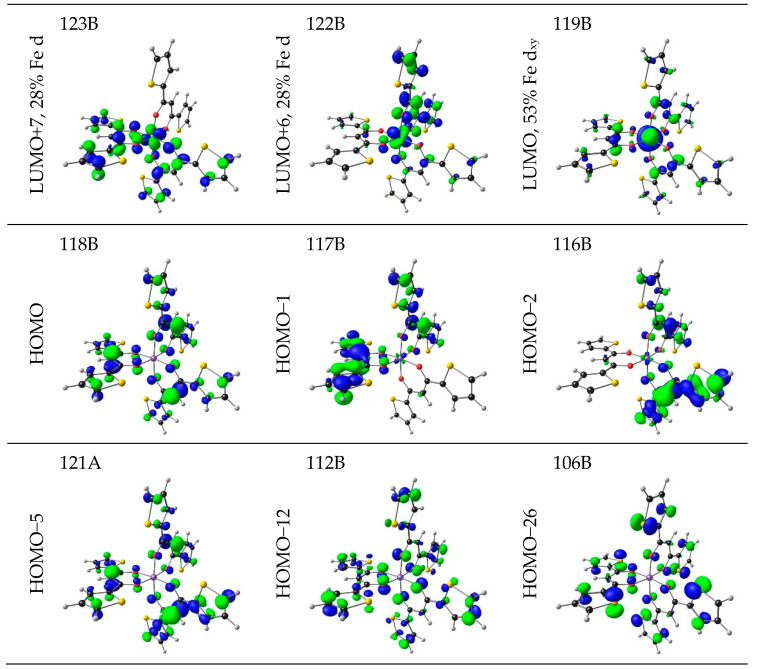
Selected Kohn−Sham molecular orbitals (MOs) of the B3LYP/CEP-121G optimized molecule 9. A contour of 0.04 Åe^−^^3^ was used for the MO plots. Color scheme used for atoms (online version): Fe (purple), O (red), C (black), S (yellow) and H (white).

To get insight into the type of charge transfer (CT) bands observed in the experimental UV-Vis spectra of 1–10, the MOs involved in the transitions were evaluated. In Table 2 and Table S2, selected calculated excitation energy (E), wavelength (λ), oscillator strengths (f) and assignments of main transitions involved in the excitation of complexes **1**, **7** and **9** are given with the MOs involved in the transitions, visualized in Figure 4, Figure 5 and Figure 6. For [Fe(acac)_3_] (1), [Fe(dbm)_3_] (7) and [Fe(dtm)_3_] (9), the maximum absorbance transition (band 2, λ_A,max_) involves excitation from ligand-based occupied MOs to mainly metal-based unoccupied MOs, thus mainly ligand-to-metal charge transfer (LMCT). Transitions at energies higher than that of the maximum absorbance transition (band 3) also have some ligand-to-ligand charge transfer (LLCT) characteristics. Similarly, band 1 and 2 transitions of complexes **2**–**8** and **10** are mainly LMCT, and band 3 are LLCT.

**Figure 7:**
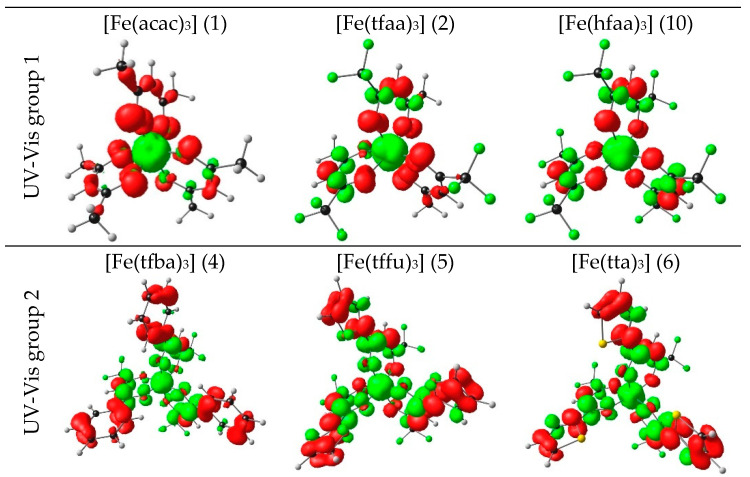

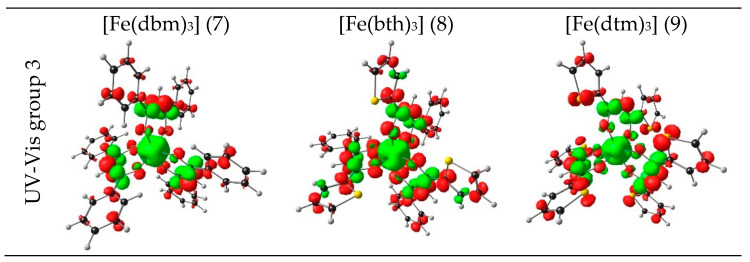
The electronic density difference plots between the ground and excited state of the maximum absorbance peak for the indicated complexes. The red surfaces indicate regions where the electron density depletes (mainly on ligand) and the green surfaces (on metal and ligand) indicate regions where the electron density increases. A contour of 0.002 (complexes **1**, **2** and **10**) or 0.001 (other complexes) Åe^−3^ was used for the electron density difference plot.

For further insight into the intramolecular charge transfer process during excitation of the maximum absorbance band, electronic density difference (EDD) plots to show the direction of the charge transfer between the ground and excited state of the maximum absorbance peak were determined and visualized in Figure 7. The region of electron density depletion (indicated with red) for all complexes is localized on the β-diketonato ligands. The region where the electron density increases (green color) is mainly on Fe(III), but also partially on the β-diketonato ligands. The EDD plots thus confirm that the maximum absorbance excitations are mainly LMCT. For the UV-Vis group 2 and 3 complexes that contain aromatic groups, the region of electron density decrease also occurs on the aromatic groups, all of pi character. Due to the symmetrical nature of the EDD plots, excitation would result in a serious intramolecular electron recombination, in agreement with the short excited state lifetimes calculated for the excited states (see Table 3 and the discussion in Section 2.1.3).

#### 2.1.3. Application as Dye to DSSC

For a complex to be used as a dye, the absorption spectra of the dye should have strong absorption peaks in the ultraviolet-visible (UV-Vis, ca. 350–750 nm) regions of the solar spectrum [20,21,22]. The experimental spectra of complexes **1**–**9** have a strong absorbance peak between 270–376 nm, with a lower energy LMCT in the 400–600 nm region. To further determine if the [Fe(β-diketonato)_3_] complexes **1**–**10** could qualify as dyes in DSSC, theoretically calculated properties for potential dyes such as light harvesting efficiency (LHE), excited state lifetime (τ in ns), HOMO energies (E_HOMO_), LUMO energies (E_LUMO_), ΔG_inject_ (eV) and ΔG_regenerate_ (eV) values are determined and presented in Table 3 for complexes **1**–**10**, for a DSSC with iodide/triiodide (I^−^/I_3_^−^) as a redox mediator (redox potential = −4.8 eV vs. vacuum, or 0.3 eV vs. NHE [23]) and anatase (TiO_2_ with *E*_CB_ = −4.0 eV vs. vacuum or −0.5 eV vs. NHE [24,25]) as a semiconductor.

One of the requirements for dyes to effective is that the unoccupied MOs (MOs to where the charge is transferred upon excitation) need to lie above the conduction band (CB) of the semiconductor (to provide a driving force for dye injection into the semiconductor), and the occupied MOs need to lie below the redox potential of the redox electrolyte (to provide a driving force for dye regeneration [26]). Although the CF_3_-containing complexes **2**, **4**–**6** and **10** have low lying LUMOs, the unoccupied MOs involved in the maximum absorbance excitation do lie sufficiently high enough to provide a driving force for dye injection: ΔG_inject_ > 0.2 eV required for a DSSC to be effective [27,28] (see Table 3). The calculated positive ΔG_inject_ and ΔG_regenerate_ values obtained for the maximum absorbance excitation for 1–10 indicate, according to definition, that electron injection and dye regeneration are spontaneous in a TiO_2_–(I^−^/I_3_^−^) DSSC. The calculated LHE (the fraction of light intensity absorbed by the dye at the specific wavelength) of 1–10 varies between 0.4 and 0.9. The LHE are the highest for complexes **5**–**9**, with one or two aromatic substituent groups on each β-diketonato ligand. The increasing π-conjugation between the β-diketonato backbone into the aromatic substituent of the donor subunit seems to enhance the LHE. The calculated excited state lifetimes (τ) of 1–10 are low (2–5 ns), and may not be sufficiently long enough to retard the charge recombination process to enhance the efficiency of the DSSCs [29]. Reported excited state lifetime values for known dyes are higher, e.g., 27 ns for CYC-B11 [30] and 11.7 ns for YD2-o-C8 [31], experimentally known efficient Ru [30] and Zn–porphyrin [32] based dye sensitizers, respectively.

### 2.2. Electrochemistry

The reduction of [Fe(β-diketonato)_3_] complexes can be considered as the acceptance of an electron into the LUMO of the complex. Since the LUMOs of 1–10 are iron-based (see Figure 5 and Figure 6 for complexes **7** and **9** as examples), the reduction is iron-based, and thus is a Fe(III/II) redox process.

#### 2.2.1. SEC

**Figure 8:**
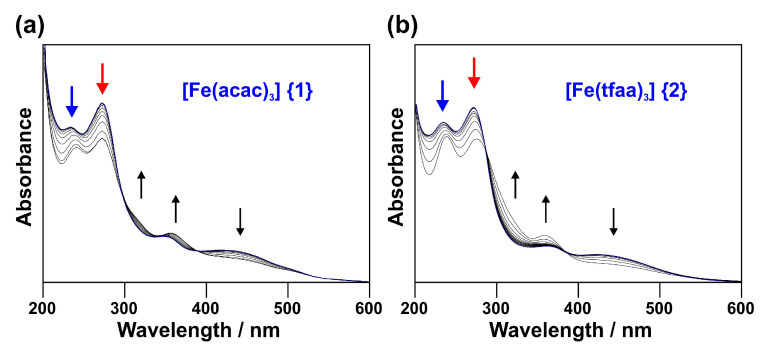
UV-Vis thin-layer spectral changes during the one-electron Fe(III)/Fe(II) reduction of (**a**) [Fe^III^(acac)_3_] complex (**1**) and (**b**) [Fe^III^(tfaa)_3_] complex (**2**). First scans are marked in blue and the peak increase/decrease is as indicated by the arrows. Red arrow highlights the decrease in the absorbance maxima of Fe(III), and the blue arrow the formation of a new absorbance maxima of Fe(II).

**Figure 9:**
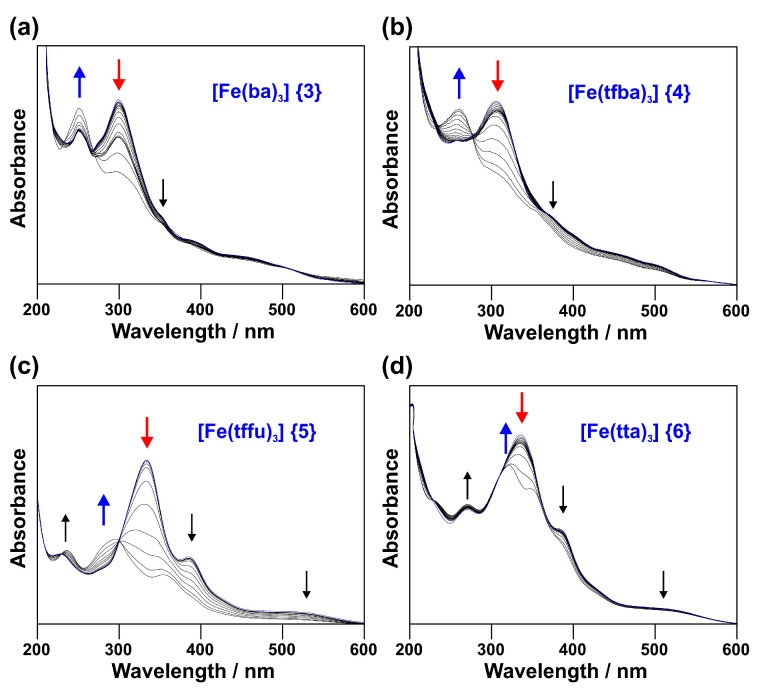
UV-Vis thin-layer spectral changes during the one-electron Fe(III)/Fe(II) reduction of (**a**) [Fe^III^(ba)_3_] complex (**3**), (**b**) [Fe^III^(tfba)_3_] complex (**4**), (**c**) [Fe^III^(tffu)_3_] complex (**5**), and (**d**) [Fe^III^(tta)_3_] complex (**6**), First scans are marked in blue and the peak increase/decrease is as indicated by the arrows. Red arrow highlights the decrease in the absorbance maxima of Fe(III), and the blue arrow the formation of a new absorbance maxima of Fe(II).

**Figure 10:**
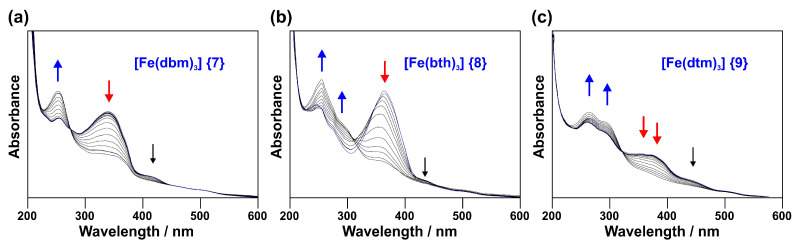
UV-Vis thin-layer spectral changes during the one-electron Fe(III)/Fe(II) reduction of (**a**) [Fe^III^(dbm)_3_] complex (**7**), (**b**) [Fe^III^(bth)_3_] complex (**8**) and (**c**) [Fe^III^(dtm)_3_] complex (**9**). First scans are marked in blue and the peak increase/decrease is as indicated by the arrows. Red arrow highlights the decrease in the absorbance maxima of Fe(III), and the blue arrow the formation of a new absorbance maxima of Fe(II).

The values of Fe(III/II) redox couple of complexes **1**–**9** have previously been reported by us [16]. To explore the UV-Vis spectral changes associated with the reduction of [Fe(β-diketonato)_3_] complexes **1**–**9**, an in situ spectroelectrochemical (SEC) study was conducted on the complexes. The spectral changes during the reduction of [Fe(β-diketonato)_3_] complexes **1**–**9** are shown in Figure 8 (UV-Vis group 1), Figure 9 (UV-Vis group 2) and Figure 10 (UV-Vis group 3), with the main changes indicated with arrows.

In Figure 8, the spectral changes during the reduction of [Fe(acac)_3_] (1) and [Fe(tfaa)_3_] (2) are shown. [Fe(acac)_3_] (1) has a strong band at 270 nm, two smaller bands at 235 and 355 nm and a shoulder at 440 nm. Upon reduction of the complex, the 235, 270 and 440 nm bands decrease in intensity and the 355 nm band increases. The 235 and 335 nm bands also have a redshift of 5 and 25 nm, respectively. Additionally, a new shoulder appears at 320 nm. Two isobestic points are visible at 295 and 390 nm. [Fe(tfaa)_3_] (2) has a strong band at 271 nm, two smaller bands at 235 and 360 nm and a shoulder at 450 nm. Upon reduction of the complex, the 235, 271 and 450 nm bands decrease in intensity and the 360 nm band increases. The 235 and 271 nm bands also both have a redshift of 4 nm. Additionally, a new shoulder appears at 315 nm. Two isobestic points are visible at 290 and 390 nm. These spectral changes are similar to that of [Fe(acac)_3_] (1). The isobestic points are indicative of chemical reversibility of the reduced species [33].

In Figure 9a, the spectral changes during the reduction of [Fe(ba)_3_] (3) are shown. This complex has a strong band at 298 nm and a smaller band at 250 nm. Upon reduction of the complex, the 298 and 250 nm bands, respectively, decrease and increase in intensity. Two isobestic points are visible at 230 and 265 nm (between 200 and 250 nm). In Figure 9b, the spectral changes during the reduction of [Fe(tfba)_3_] (4) are shown. This complex has a strong band at 304 nm and a shoulder at 259 nm. Upon reduction of the complex, the 304 and 259 nm bands, respectively, decrease and increase in intensity. Two isobestic points are visible at 230 nm and 275 nm (between 200 and 300 nm). In Figure 9c, the spectral changes during the reduction of [Fe(tffu)_3_] (5) are shown. This complex has a strong band at 333 nm, two smaller bands at 230 and 385 nm and a shoulder at ~520 nm. Upon reduction of the complex, the 333, 385 and ~520 nm bands disappear completely. A new strong band at 295 nm and a shoulder at 350 nm appear. The 230 nm band increases with a redshift of 5 nm. Two isobestic points are visible at 230 and 300 nm. In Figure 9d, the spectral changes during the reduction of [Fe(tta)_3_] (6) are shown. This complex has a strong band at 333 nm, a smaller band at 270 nm and a shoulder at 385 nm. Upon reduction of the complex, the 333 nm band decreases in intensity to form a band at 320 nm with a shoulder at 350 nm. The shoulder at 385 nm also decreases in intensity. The band at 270 nm slightly increases in intensity and a new shoulder at 230 nm appears. Two isobestic points are visible at 225 and 300 nm.

In Figure 10a, the spectral changes during the reduction of [Fe(dbm)_3_] (7) are shown. This complex has a strong band at 336 nm, a smaller band at 251 nm and a shoulder at 415 nm. Upon reduction of the complex, the 336 nm band decreases in intensity and the shoulder at 415 nm disappears completely. The 251 nm band increases in intensity with a slight blueshift of 3 nm. One isobestic point is visible at 270 nm. In Figure 10b, the spectral changes during the reduction of [Fe(bth)_3_] (8) are shown. This complex has a strong band at 361 nm, a smaller band at 245 nm and a shoulder at 275 nm. Upon reduction of the complex, the 361 nm band decreases in intensity. The 245 nm band initially slightly decreases with a redshift of 10 nm, and then strongly increases at 255 nm. The shoulder at 275 nm increases with a redshift of 25 nm. One isobestic point is visible at ~300 nm. In Figure 10c, the spectral changes during the reduction of [Fe(dtm)_3_] (9) are shown. This complex has a band at 260 nm and four shoulders at 295, 350, 375 and 445 nm. Upon reduction of the complex, the 350, 375 and 445 nm shoulders decrease in intensity and the 260 nm band with the 295 nm shoulder increases in intensity. One isobestic point is visible at 320 nm.

**Figure 11:**
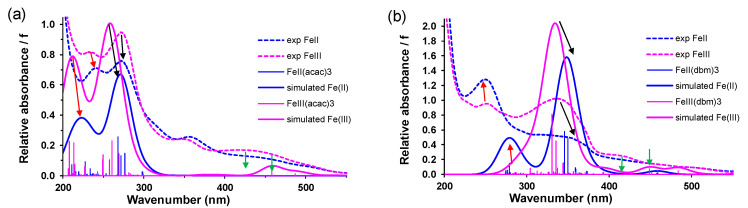
Experimental (dotted lines) and B3LYP TDDFT-calculated (simulated spectra in smooth solid lines and calculated oscillators shown as thin vertical lines) UV-Vis of (**a**) [Fe^III^(acac)_3_] (magenta) and [Fe^II^(acac)_3_]^−^ (blue) and (**b**) [Fe^III^(dbm)_3_] (magenta) and [Fe^II^(dbm)_3_]^−^ (blue). Selected differences between the spectra of the Fe(II) and Fe(III) complexes are highlighted by arrows.

In comparing the spectral changes during the one-electron Fe(III)/Fe(II) reduction of complexes **1**–**9**, the maximum absorbance peak of all the Fe(III) complexes decreases (indicated with red arrows in the Figure 8, Figure 9 and Figure 10). A new maximum absorbance peak associated with Fe(II) forms at a lower wavelength (indicated with blue arrows in the Figure 8, Figure 9 and Figure 10). For the thienyl- and furyl-containing complexes **5**, **6**, **8** and **9**, two new absorbance peaks associated with Fe(II) form at the lower wavelength. The absorbance peak associated with Fe(II) that forms at the lower wavelength decreases in intensity for the UV-Vis group 1 complexes, while it increases in intensity for the UV-Vis group 2 and group 3 complexes. This peak might be related to the pi orbitals on the aromatic groups of complexes **3**–**9**. In Figure 11, the experimental and TDDFT UV-Vis of [Fe^III^(β-diketonato)_3_] and [Fe^II^(β-diketonato)_3_]^−^ are compared for β-diketonato = acac (complex **1**, without any aromatic group) and dbm (complex **9**, containing aromatic groups). Both the experimental and calculated spectra of [Fe(acac)_3_] show similar features (Figure 11a), namely: (i) the two strong absorbance peaks in the 200–300 nm region decrease in intensity and redshift upon reduction of Fe(III), and (ii) the lower energy, higher wavelength band above 400 nm disappears upon reduction of Fe(III). Only the redshift of the small experimental peak at ca. 350 nm could not be identified in the calculated spectra. For [Fe(dbm)_3_], the calculated spectra reproduced the main features of the experimental spectra (Figure 11b), namely: (i) the strong absorbance peaks below 300 nm increase in intensity and blueshift upon reduction of Fe(III), (ii) the strong absorbance peaks at ca. 350 nm decrease in intensity and redshift upon reduction of Fe(III), and (iii) the lower energy, higher wavelength band above 400 nm disappears upon reduction of Fe(III).

The main features of the spectral changes during the one-electron Fe(III)/Fe(II) reduction are thus:

(i) The maximum absorbance peak is at a higher wavelength of all the Fe(III) complexes that decrease (indicated with red arrows in the Figure 8, Figure 9 and Figure 10) and;
(ii) The new maximum absorbance peak is associated with Fe(II) that forms at a lower wavelength (indicated with blue arrows in the Figure 8, Figure 9 and Figure 10).

**Figure 12:**
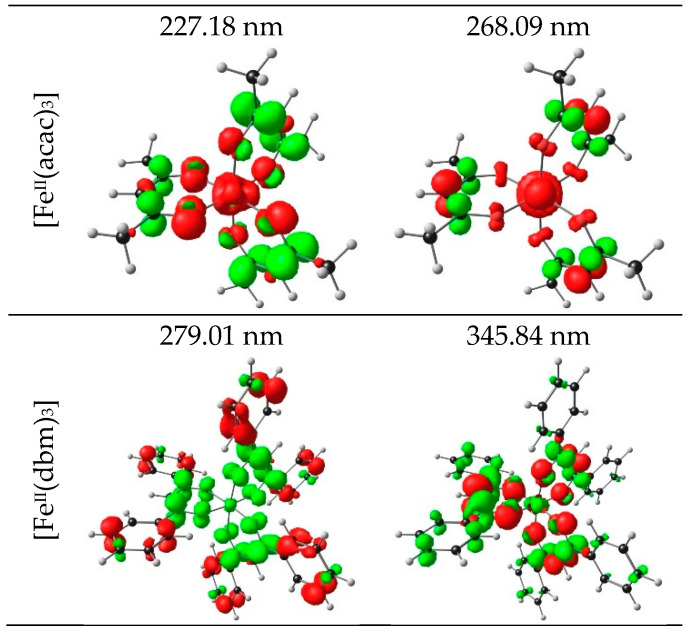
The electronic density difference plots between the ground and excited state of the maximum absorbance peaks for the indicated Fe(II) complexes. The red surfaces indicate the regions where the electron density depletes and the green surfaces indicate the regions where the electron density increases. A contour of 0.002 Åe^−3^ was used for the electron density difference plot.

The EDD plots between the ground and excited state of these two maximum absorbance peaks were determined for complex **1** (representative of non-aromatic-containing complexes) and complex **7** (representative of complexes containing aromatic groups), and illustrated in Figure 12. For [Fe^II^(acac)_3_]^−^, the region of electron density depletion upon excitation (indicated with red) is on Fe(II) and the ligands. The HOMO of [Fe^II^(acac)_3_]^−^ is of Fe-d character (the Fe-d-based MO that accepted the electron upon reduction of Fe(III)), and this electron density depletes upon Fe(II) excitation, leading to the decrease of intensity of both absorbance maxima peaks (at 227.18 and 268.09 nm) compared to Fe(III) (Figure 11a).

For [Fe^II^(dbm)_3_]^−^, however, the region of electron density depletion upon excitation (indicated with red in Figure 12) is mainly on the aromatic groups for the lower wavelength absorbance maxima (279.01 nm), and on the ligand (with a small fraction on Fe(II)) for the higher wavelength absorbance maxima (345.84 nm). Both peaks thus involve mainly intra ligand electron transfer, leading to an increase of the lower wavelength absorbance maxima (279.01 nm), and decrease of higher wavelength absorbance maxima peak (345.84 nm) (Figure 11b). The increase in intensity of the lower wavelength absorbance maxima of complexes **3**–**9**, containing aromatic groups, are thus related to charge transfer from the aromatic groups upon excitation.

#### 2.2.2. Application as Redox Mediator in DSSC

**Figure 13:**
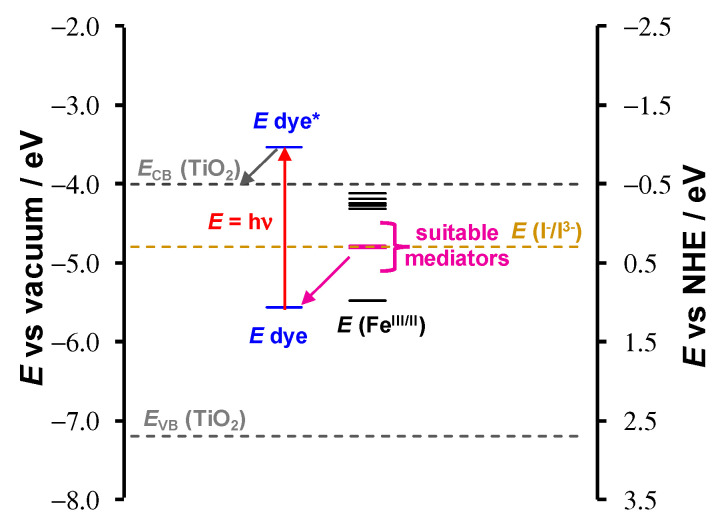
Energy level diagram of the redox values of the dye LEG4 (blue, data from references [37,38] in CH_3_CN) and the Fe(III/II) redox potentials of complexes **1**–**10** (black or purple). The redox potential of complexes **2**, **4**, **5** and **6**, suitable for use as redox mediators in a DSSC with an anatase TiO_2_ semiconductor, are indicated in purple. The redox potential of I^−^/I_3_^−^ (light brown) is shown for comparative purposes.

In DSSCs, the redox mediator needs to regenerate the oxidized dye. The well-known (I_3_^−^/I^−^) redox couple has been used as redox couple in DSSC for many years [34]. Any other redox couple that is considered may have a redox potential up to 0.5 V more positive than that of (I_3_^−^/I^−^) [34]. The Fe(III/II) redox couple [16] of complexes **1**–**10** are compared to the redox potential of the (I_3_^−^/I^−^) redox couple (0.3 V vs. NHE [23]) in Figure 13. Complexes 1–10 can be grouped into three groups according to the experimentally measured redox value of the Fe(III/II) redox couple:

Redox group 1: *E*^0′^ < −0.18 V vs. NHE, complexes **1**, **3** and **7**–**9**, containing no CF_3_ substituent groups on the β-diketonato ligands.

Redox group 2: *E*^0′^ = 0.2–0.3 V vs. NHE, complexes **2** and **4**–**6**, containing one CF_3_ substituent groups on the β-diketonato ligands.

Redox group 3: *E*^0′^ > 0.9 V vs. NHE, complex **10**, containing two CF_3_ substituent groups on the β-diketonato ligands.

Considering a DSSC with the organic LEG4 dye [35,36] (redox potential = 1.07 V vs. NHE in CH_3_CN [37], excited state = −0.97 vs. NHE [38]) adsorbed onto a film of TiO_2_ semiconductor, only the redox potentials of redox group 2 are suitable for use as a redox mediator. However, with another dye and semiconductor, more complexes may qualify as redox mediators in DSSC [39]. For example, [Fe(acac)_3_] complex **1** showed promising results as a redox mediator in p-type DSSCs in conjunction with a perylene–thiophene–triphenylamine sensitizer and NaO as a semiconductor [2].

## 3. Materials and Methods

### 3.1. General

Complexes 1–10 were synthesized and characterized as reported in the literature [16]. Characterization data agree with reported data and are provided in the supplementary information. UV-Vis spectra were recorded on a Varian Cary 50 Conc ultra-violet/visible spectrophotometer.

Spectroelectrochemical measurements were performed on 0.003 mol dm^−3^ solutions of the complex dissolved in CH_3_CN as a solvent, containing 0.200 mol dm^−3^ tetra-n-butylammonium hexafluorophoshate as the supporting electrolyte. An optically transparent thin layer electrochemical (OTTLE) Omni cell system, fitted with NaCl liquid Omni windows, was filled with the solution. The OTTLE cell was connected to a Cary 50 Conc ultra-violet/visible spectrophotometer, as well as a BAS100B electrochemical analyzer (linked to a personal computer utilizing the BAS100W Version 2.3 software). Spectra were collected on the spectrophotometer every 5 min between 200–600 nm (for 90 min or till no spectral changes occurred) while scanning at a rate of 500 μV s^−1^ on the electrochemical analyzer from the resting potential of the iron(III) complex until 0.5 V after the Fe(III/II) reduction potential of the complex. Spectral (absorbance vs. wavelength) data were collected, and were exported as csv data and imported into Microsoft Office Excel for analysis. Spectral processing and visualization were also done with Microsoft Office Excel.

### 3.2. DFT Methods

Density functional theory (DFT) calculations were performed on the molecules using the Gaussian 16 computational chemistry software package [40]. Optimization of the molecules was performed using the (i) B3LYP [41,42], (ii) M06 [43], (iii) PBEh1PBE, (iv) CAM-B3LYP [44] and (v) PBE1PBE functionals, in combination with the CEP-121G [45], aug-cc-pVDZ [46,47], cc-pVTZ [46,47,48,49,50,51], def2tzvpp [52], LanL2DZ [53,54,55] and SDD [56] basis sets. Where indicated, the Grimme’s D3 dispersion correction was used [57]. Optimizations were performed in CH_3_CN as solvent (*ε*_r_ = 37.5), using the implicit solvent polarizable continuum model (PCM) [58] that uses the integral equation formalism variant (IEFPCM) [59]. Frequency and time-dependent density functional theory (TDDFT) calculations were performed on the same level of theory. Multiwfn [60] was used to create the cube files for the electron and hole for the electronic density difference (EDD) plots between the ground and a specific excited state. The input coordinates for the compounds were constructed using Chemcraft [61]. The results of the TDDFT calculations and the character and energy of the (Kohn−Sham) molecular orbitals (MOs) were obtained from the DFT output files and visualized using Chemcraft or Microsoft Office Excel. The driving force for dye regeneration (ΔG_regenerate_) and injection (ΔG_inject_) into a semiconductor of a DSSC was calculated as described in the literature [19], with positive values indicating a spontaneous process. DFT calculations previously performed by us [16,17], in agreement with the experiment [62], showed that tris(β-diketonato)iron complexes are high spin; thus, all calculations for Fe(III) were conducted with S = 5/2 and Fe(II) with S = 2.

## 4. Conclusions

The experimental UV-Vis absorbance maxima of tris(β-diketonato)iron(III) complexes redshifts with more aromatic substituent groups on the β-diketonato ligand in tris(β-diketonato)iron(III) complexes. The experimental reduction potential of tris(β-diketonato)iron(III) complexes become more positive with more CF_3_ substituent groups on the β-diketonato ligand in tris(β-diketonato)iron(III) complexes. DFT calculations show that the absorbance maxima peaks in the UV-Vis spectrum are mainly LMCT band, and that the reduction of the complexes are metal-based. DFT calculations further show that some of the tris(β-diketonato)iron(III) exhibit properties that mean they can be considered as dye sensitizers or as redox mediators in DCCS.

## Figures and Tables

**Table 1:** Experimental and B3LYP/CEP-121G/CH_3_CN-, CAM-B3LYP/CEP-121G/CH_3_CN- and M06/CEP-121G/CH_3_CN-calculated absorbance maxima wavelength (nm) of [Fe(β-diketonate)_3_] complexes **1**–**10**. The density functional theory (DFT) calculated electronic energy difference (ΔE in eV), zero-point-corrected electronic energies difference (ΔZEE in eV) and free energy difference (ΔG in eV) between the *fac*- and *mer*-isomers are also given.

No.	β-Diketone	λ_A,max,exp_	λ_A,max,calc_	λ_A,max,calc_	λ_A,max,calc_	ΔE			ΔZEE		ΔG	
		Exp	B3LYP	M06	CAM-B3LYP	B3LYP	M06	CAM-B3LYP	B3LYP	M06	B3LYP	M06
1	acac	270	260.3	254.9	255.3	-	-	-	-	-	-	-
2	tfaa *fac*	271	261.9	256.0	261.0	0.004	0.017	0.005	0.000	0.005	0.000	0.001
	tfaa *mer*	271	262.1	256.6	261.5	0.000	0.000	0.000	0.003	0.000	0.091	0.000
3	ba *fac*	298	303.1	301.3	282.2	0.001	0.043	0.000	0.000	0.042	0.000	0.000
	ba *mer*	298	304.2	309.9	287.0	0.000	0.000	0.000	0.001	0.000	0.015	0.021
4	tfba *fac*	304	311.0	309.8	290.6	0.008	0.051	0.008	0.004	0.034	0.001	0.000
	tfba *mer*	304	318.6	320.2	307.8	0.000	0.000	0.000	0.000	0.000	0.000	0.025
5	tffu *fac*	333	321.0	317.0	297.8	0.000	0.010	0.000	0.002	0.008	0.007	0.141
	tffu *mer*	333	333.0	327.1	325.5	0.000	0.000	0.001	0.000	0.000	0.000	0.000
6	tta *fac*	333	329.0	325.7	321.5	0.002	0.045	0.000	0.002	0.033	0.011	0.011
	tta *mer*	333	339.2	339.8	333.1	0.000	0.000	0.000	0.000	0.000	0.000	0.000
7	dbm	336	330.7	341.8	302.2	-	-	-	-	-	-	-
8	bth *fac*	361	346.6	346.6	326.8	0.000	0.043	0.000	0.004	0.039	0.039	0.036
	bth *mer*	361	347.0	351.4	327.2	0.000	0.000	0.000	0.000	0.000	0.000	0.000
9	dtm	376	375.8	358.7	333.7	-	-	-	-	-	-	-
10	hfaa	-	278.3	274.0	268.1	-	-	-	-	-	-	-
	AD ^a^		7.8	11.0	18.4							
	MAD ^b^		3.8	4.3	11.6							

(^a^) AD = Average deviation from experiment. (^b^) MAD = Mean absolute deviation from experiment.

**Table 2:** B3LYP/CEP-121G-calculated excitation energy (E), absorbance maximum wavelength (λ), oscillator strengths (f) and assignments of main transitions involved in the indicated excitations of complexes **1**, **7** and **9**.

Complex	E/eV	λ/nm	f	from	to	% Contribution	Assignment
1	4.76	260.34	0.2286	HOMO−16	LUMO+2	6.09	LMCT
				HOMO−11	LUMO+6	6.71	LMCT
				HOMO−11	LUMO+9	25.49	LMCT
				HOMO−10	LUMO+8	25.70	LMCT
	4.76	260.33	0.2279	HOMO−16	LUMO+1	6.12	LMCT
				HOMO−11	LUMO+7	6.69	LMCT
				HOMO−11	LUMO+8	25.79	LMCT
				HOMO−10	LUMO+6	6.75	LMCT
				HOMO−10	LUMO+9	25.65	LMCT
7	3.75	330.68	0.8092	HOMO−3	LUMO+5	3.71	LLCT
				HOMO−1	LUMO+3	4.02	LLCT
				HOMO−35	LUMO+2	3.61	LMCT
				HOMO−34	LUMO+1	3.55	LMCT
				HOMO−27	LUMO	4.28	LMCT
				HOMO−18	LUMO+1	3.62	LMCT
				HOMO	LUMO+6	20.48	LMCT
9	3.30	375.76	0.5211	HOMO−26	LUMO	17.80	LMCT
				HOMO−12	LUMO	7.43	LMCT
				HOMO−2	LUMO+6	7.30	LMCT
				HOMO−2	LUMO+7	6.41	LMCT
				HOMO−1	LUMO+7	6.99	LMCT

LMCT = ligand-to-metal charge transfer, LLCT = ligand-to-ligand charge transfer, HOMO = highest occupied molecular orbital, LUMO = lowest unoccupied molecular orbital.

**Table 3:** B3LYP/CEP-121G-calculated excitation energy (E), absorbance maximum wavelength (λ), oscillator strengths (f), light harvesting efficiency (LHE), excited state lifetime (τ) and HOMO and LUMO energies of complexes **1**–**10**. DFT-calculated ΔG_inject_ (related to λ_A,max_) and ΔG_regenerate_ for a DSSC with (I^−^/I_3_^−^) as the redox mediator and anatase TiO_2_ as the semiconductor are also indicated.

No	Ligand	λ_A,max_	f	E	LHE	τ	E_LUMO_	E_HOMO_	ΔG_inject_	ΔG_regenerate_
		nm		eV		ns	eV	eV	eV	eV
1	acac	260.34	0.23	4.76	0.41	4	−3.196	−6.552	2.21	1.75
2	tfaa *fac*	261.86	0.30	4.73	0.49	3	−4.117	−7.387	1.35	2.59
	tfaa *mer*	262.12	0.33	4.73	0.53	3	−4.114	−7.374	1.36	2.57
3	ba *fac*	303.05	0.39	4.09	0.59	4	−3.263	−6.463	1.63	1.66
	ba *mer*	304.16	0.30	4.08	0.50	5	−3.265	−6.459	1.62	1.66
4	tfba *fac*	310.95	0.53	3.99	0.71	3	−4.064	−7.172	0.81	2.37
	tfba *mer*	318.63	0.49	3.89	0.67	3	−4.064	−7.126	0.77	2.33
5	tffu *fac*	320.96	0.93	3.86	0.88	2	−4.075	−7.048	0.82	2.25
	tffu *mer*	332.98	0.67	3.72	0.78	2	−4.080	−6.972	0.75	2.17
6	tta *fac*	328.98	0.90	3.77	0.88	2	−4.066	−7.032	0.74	2.23
	tta *mer*	339.16	0.68	3.66	0.79	3	−4.065	−6.965	0.69	2.16
7	dbm	330.68	0.81	3.75	0.84	2	−3.330	−6.373	1.38	1.57
8	bth *fac*	346.64	0.80	3.58	0.84	2	−3.394	−6.312	1.26	1.51
	bth *mer*	346.96	0.37	3.57	0.57	5	−3.391	−6.311	1.26	1.51
9	dtm	375.76	0.52	3.30	0.70	4	−3.330	−6.373	0.93	1.57
10	hfaa	278.26	0.22	4.46	0.40	5	−5.122	−8.199	0.26	3.40

## Data Availability

The data presented in this study are available in the Supplementary Materials.

## Supplementary Materials

The following supporting information can be downloaded at: https://www.mdpi.com/article/10.3390/molecules27123743/s1, Section S1: Table S1: TDDFT-calculated λ_max_ and the corresponding oscillator strength (f) data of [Fe(acac)_3_], complex **1**, using the indicated DFT functional and basis set combinations. λmax(experimental) = 270 nm; Table S2: B3LYP/CEP-121G-calculated excitation energy (E), absorbance maximum wavelength (λ), oscillator strengths (f) and assignments of main transitions involved in the indicated excitations of complexes **1** and **9**. (a: LUMO+3 on ligand, extended onto Th); Section S2: Characterization data of complexes; Section S3: Optimized coordinates of the DFT calculations.

## Sample Availability

Samples of the compounds are not available from the authors.
