# Supplementary material for: UV-Vis Spectroscopy, Electrochemical and DFT Study of Tris(β-diketonato)iron(III) Complexes with Application in DSSC: Role of Aromatic Thienyl Groups
Source: Molecules. 2022 Jun 10;27(12):3743. doi: 10.3390/molecules27123743 (PMC9227534; doi:10.3390/molecules27123743)
Supplement: Supplementary file 1 [file molecules-27-03743-s001.zip › molecules-1741028-supplementary.pdf]

UV-Vis Spectroscopy, Electrochemical and DFT Study of Tris(β-diketonato)iron(III) Complexes with Application in DSSC: Role of Aromatic Thienyl Groups

*Marrigje M. Conradie **

Department of Chemistry, University of the Free State, P.O. Box 339, Bloemfontein, 9300, South Africa

Contact author details:

Name: Marrigje M. Conradie, Tel: ++27-51-4019898, email: conradiemm@ufs.ac.za

0000-0001-8104-7684 (MM Conradie)

Supporting information

Table of Contents

Section S1: Tables 3
Section S2: Characterization data 5
$[Fe(acac)_3]$ (**1**) 5
$[Fe(tfaa)_3]$ (**2**) 5
$[Fe(ba)_3]$ (**3**) 5
$[Fe(tfba)_3]$ (**4**) 5
$[Fe(tffu)_3]$ (**5**) 5
$[Fe(tta)_3]$ (**6**) 5
$[Fe(dbm)_3]$ (**7**) 5
$[Fe(bth)_3]$ (**8**) 6
$[Fe(dtm)_3]$ (**9**) 6
$[Fe(hfaa)_3]$ (**10**) 6
Section S3: Optimized Cartesian coordinates (Å) 7
$[Fe(acac)_3]$ 7
$[Fe(tfaa)_3]$ *fac* 8
[Fe(tfaa)3] mer 9

[Fe(ba)$_3$] *fac* 9
[Fe(ba)$_3$] *mer* 11
[Fe(tfba)$_3$] *fac* 12
[Fe(tfba)$_3$] *mer* 14
[Fe(tffu)$_3$] *fac* 15
[Fe(tffu)$_3$] *mer* 16
[Fe(tta)$_3$] *fac* 17
[Fe(tta)$_3$] *mer* 18
[Fe(dbm)$_3$] 20
[Fe(bth)$_3$] *fac* 21
[Fe(bth)$_3$] *mer* 23
[Fe(dtm)$_3$] 25
[Fe(hfaa)$_3$] 26

Section S1: Tables

Table S1. TDDFT calculated λ_{max} and the corresponding oscillator strength (f) data for of $[Fe(acac)_3]$, complex 1, using the indicated DFT functional and basis set combinations. λ_{max}(experimental) = 270 nm

Functional	Basis set	λ_{max}	f
B3LYP	aug-cc-pVDZ	255.83	0.2800
	cc-pVTZ	218.78	0.3186
	CEP-121G	260.35	0.2284
	def2tzvpp	219.11	0.3322
	LanL2DZ	224.75	0.5270
	SDD	254.31	0.2377
M06	aug-cc-pVDZ	247.73	0.2249
	cc-pVTZ	201.45	0.3101
	CEP-121G	254.90	0.2174
	def2tzvpp	202.47	0.2978
	LanL2DZ	218.28	0.2843
	SDD	250.68	0.1653
PBEh1PBE	aug-cc-pVDZ	207.01	0.3331
	cc-pVTZ	204.55	0.3389
	CEP-121G	244.50	0.3553
	def2tzvpp	204.88	0.3408
	LanL2DZ	216.19	0.4266
	SDD	249.98	0.2465
PBE1PBE	aug-cc-pVDZ	206.42	0.3295
	cc-pVTZ	203.86	0.3322
	CEP-121G	244.26	0.3588
	def2tzvpp	204.18	0.3339
	LanL2DZ	216.12	0.4422
	SDD	239.32	0.2447
B3LYP geometry, CAM-B3LYP TDDFT	CEP-121G	255.33	0.3855
B3LYP-D3	CEP-121G	257.68	0.3391
PBE-D3 geometry, B3LYP TDDFT	CEP-121G	215.87	0.2821
PBE geometry, B3LYP TDDFT	CEP-121G	216.55	0.2742

Table S2. B3LYP/CEP-121G calculated excitation energy (*E*), absorbance maximum wavelength (λ), oscillator strengths (f), assignments of main transitions involved in the indicated excitations of complexes 1 and 9. (a: LUMO+3 on ligand, extended onto Th)

Coml	E/eV	λ/nm	f	from	to	% contribution	assignment
1	2.46	503.17	0.0032	HOMO−5	LUMO	82.11	LMCT
	2.55	486.81	0.0123	HOMO−4	LUMO	8.72	LMCT
				HOMO	LUMO+1	73.26	LMCT
				HOMO	LUMO+2	9.90	LMCT
	2.71	458.13	0.0311	HOMO−5	LUMO+2	44.03	LMCT
				HOMO−4	LUMO+1	41.30	LMCT
	2.71	458.08	0.0313	HOMO−5	LUMO+1	41.91	LMCT
				HOMO−4	LUMO+2	43.42	LMCT
	4.76	260.34	0.2286	HOMO−16	LUMO+2	6.09	LMCT
				HOMO−11	LUMO+6	6.71	LMCT
				HOMO−11	LUMO+9	25.49	LMCT
				HOMO−10	LUMO+8	25.70	LMCT
	4.76	260.33	0.2279	HOMO−16	LUMO+1	6.12	LMCT
				HOMO−11	LUMO+7	6.69	LMCT
				HOMO−11	LUMO+8	25.79	LMCT
				HOMO−10	LUMO+6	6.75	LMCT
				HOMO−10	LUMO+9	25.65	LMCT
	5.82	213.09	0.2173	HOMO−13	LUMO+4	7.10	LLCT
				HOMO−13	LUMO+5	6.73	LLCT
				HOMO−12	LUMO+4	6.90	LLCT
				HOMO−12	LUMO+5	7.25	LLCT
				HOMO−24	LUMO	8.56	LMCT
				HOMO−15	LUMO+9	13.44	LMCT
				HOMO−14	LUMO+8	12.95	LMCT
	5.96	208.00	0.2256	HOMO−13	LUMO+4	17.40	LLCT
				HOMO−13	LUMO+5	16.56	LLCT
				HOMO−12	LUMO+4	16.27	LLCT
				HOMO−12	LUMO+5	17.26	LLCT
				HOMO−24	LUMO	9.95	LMCT
9	1.90	651.25	0.0163	HOMO−5	LUMO+3[a]	7.60	LLCT
				HOMO	LUMO	57.52	LMCT
	2.38	521.61	0.0355	HOMO−2	LUMO+2	18.30	LMCT
				HOMO−1	LUMO+1	18.76	LMCT
				HOMO	LUMO+2	28.33	LMCT
	2.38	521.38	0.0364	HOMO−2	LUMO+1	19.52	LMCT
				HOMO−1	LUMO+2	18.82	LMCT
				HOMO	LUMO+1	26.88	LMCT
	2.55	486.30	0.1004	HOMO−5	LUMO+3	6.81	LMCT
				HOMO−2	LUMO+2	19.46	LMCT
				HOMO−1	LUMO+1	18.52	LMCT
	3.30	375.76	0.5211	HOMO−26	LUMO	17.80	LMCT
				HOMO−12	LUMO	7.43	LMCT
				HOMO−2	LUMO+6	7.30	LMCT
				HOMO−2	LUMO+7	6.41	LMCT
				HOMO−1	LUMO+7	6.99	LMCT

Section S2: Characterization data

[Fe(acac)$_3$] (1)
Yield 73%. M.p. 180-182 °C). UV: λ_{max} 270 nm, ε_{max} 25842 mol^{-1}.dm^3.cm^{-1} (CH_3CN). MS Calcd. ([M]$^-$, positive mode): *m/z* 352.2. Found: *m/z* 351.9. Anal. Calcd. for $FeC_{15}H_{21}O_6$: C, 51.01; H, 5.99. Found: C, 50.42; H, 5.91.

[Fe(tfaa)$_3$] (2)
Yield 91%. M.p. 113.5-116.1 °C. UV: λ_{max} 271 nm, ε_{max} 48996 mol^{-1}.dm^3.cm^{-1} (CH_3CN). MS Calcd. ([M]$^-$, negative mode): *m/z* 514.1. Found: *m/z* 513.5. Anal. Calcd. for $FeC_{15}H_{12}O_6F_9$: C, 34.98; H, 2.35. Found: C, 34.48; H, 2.30.

[Fe(ba)$_3$] (3)
Yield 84%. Colour: red-orange. M.p. 218.5-220.3 °C. UV: λ_{max} 298 nm, ε_{max} 40256 mol^{-1}.dm^3.cm^{-1} (CH_3CN). MS Calcd. ([M], positive mode): *m/z* 539.4. Found: *m/z* 539.1. Anal. Calcd. for $FeC_{30}H_{27}O_6$: C, 66.80; H, 5.05. Found: C, 66.46; H, 5.10.

[Fe(tfba)$_3$] (4)
Yield 96%. Colour: orange. M.p. 58.4-60.1 °C. UV: λ_{max} 304 nm, ε_{max} 30788 mol^{-1}.dm^3.cm^{-1} (CH_3CN). MS Calcd. ([M], negative mode): *m/z* 701.3. Found: *m/z* 701.1. Anal. Calcd. for $FeC_{30}H_{18}O_6F_9$: C, 51.38; H, 2.59. Found: C, 50.66; H, 2.53.

[Fe(tffu)$_3$] (5)
Yield 92%. Colour: dark red. M.p. 205.0-208.0 °C. UV: λ_{max} 333 nm, ε_{max} 53476 mol^{-1}.dm^3.cm^{-1} (CH_3CN). MS Calcd. ([M], positive mode): *m/z* 671.2. Found: *m/z* 671.1. Anal. Calcd. for $FeC_{24}H_{12}O_9F_9$: C, 42.95; H, 1.80. Found: C, 42.91; H, 1.83.

[Fe(tta)$_3$] (6)
Yield 78%. Colour: dark red. M.p. 158.0-161.0 °C. UV: λ_{max} 333 nm, ε_{max} 48461 mol^{-1}.dm^3.cm^{-1} (CH_3CN). MS Calcd. ([M], negative mode): *m/z* 719.4. Found: *m/z* 718.9. Anal. Calcd. for $FeC_{24}H_{12}S_3O_6F_9$: C, 40.07; H, 1.68. Found: C, 39.51; H, 1.70.

[Fe(dbm)$_3$] (7)
Yield 71%. M.p. 264-265.2 °C. UV: λ_{max} 336 nm, ε_{max} 48608 mol^{-1}.dm^3.cm^{-1} (CH_3CN). MS Calcd. ([M]$^-$, positive mode): *m/z* 725.6. Found: *m/z* 725.3. Anal. Calcd. for $FeC_{45}H_{33}O_6$: C, 74.49; H, 4.58. Found: C, 75.29; H, 4.51.

[Fe(bth)$_3$] (8)

Yield 85%. Colour: red-brown. M.p. 220.0-222.0 °C. UV: λ_{max} 361 nm, ε_{max} 30956 mol^{-1}.dm^3.cm^{-1} (CH_3CN). MS Calcd. ([M], positive mode): *m/z* 743.7. Found: *m/z* 743.2. Anal. Calcd. for $FeC_{39}H_{27}S_3O_6$: C, 62.99; H, 3.66. Found: C, 61.44; H, 3.71.

[Fe(dtm)$_3$] (9)

Yield 39%. Colour: black. M.p. 63.7-66.4 °C. UV: λ_{max} 260 nm, ε_{max} 35301 mol^{-1}.dm^3.cm^{-1} (CH_3CN). This complex could not be ionized for MS analysis. Anal. Calcd. for $FeC_{33}H_{21}S_6O_6$: C, 52.03; H, 2.78. Found: C, 51.27; H, 2.71.

[Fe(hfaa)$_3$] (10)

Yield 51%. Colour: Yellow-red. M.p. 56-58 °C. MS Calcd. ([M]$^-$, negative mode): *m/z* 677.0. Found: *m/z* 676.8. Anal. Calcd. for $FeC_{15}H_3O_6F_{18}$: C, 26.61; H, 0.45. Found: C, 26.17; H, 0.51.

Section S3: Optimized Cartesian coordinates (Å)

All compounds were optimized the B3LYP functional and the CEP-121G basis set.

[Fe(acac)$_3$]

```
43
symmetry c1
Fe        0.000518000      -0.000244000       0.000512000
O         1.531349000       0.620089000       1.162062000
O         1.425354000      -0.833785000      -1.162297000
O        -0.227972000      -1.635935000       1.162307000
O        -1.435498000      -0.816513000      -1.161142000
O        -1.299345000       1.015590000       1.164667000
O         0.007978000       1.650021000      -1.162781000
C        -1.814827000       2.226020000       1.064504000
C        -1.513338000       3.117814000       0.000101000
C        -0.625257000       2.803292000      -1.063622000
C         2.837295000       0.460994000       1.061367000
C         3.458064000      -0.248520000      -0.001804000
C         2.740807000      -0.862425000      -1.063737000
C        -1.019564000      -2.686792000       1.062350000
C        -1.945854000      -2.868796000       0.000286000
C        -2.119077000      -1.940686000      -1.061633000
C         3.475840000      -1.604563000      -2.169409000
H         4.558435000      -1.594196000      -2.018421000
H         3.126205000      -2.644317000      -2.209920000
H         3.241905000      -1.145166000      -3.138642000
C         3.672301000       1.090087000       2.166175000
H         3.376819000       0.667946000       3.135557000
H         4.742146000       0.925740000       2.013451000
H         3.474352000       2.168991000       2.207839000
C        -0.352855000       3.810214000      -2.170629000
H         0.722321000       4.027167000      -2.214056000
H        -0.636241000       3.377251000      -3.138842000
H        -0.902714000       4.742721000      -2.018917000
C        -2.774730000       2.634956000       2.171270000
H        -3.610711000       1.924818000       2.214054000
H        -2.259675000       2.589079000       3.139668000
H        -3.166740000       3.644075000       2.019816000
C        -3.130740000      -2.205325000      -2.166220000
H        -2.617246000      -2.231257000      -3.136187000
H        -3.662679000      -3.148310000      -2.015461000
H        -3.856627000      -1.382776000      -2.204890000
C        -0.891455000      -3.724880000       2.166682000
H        -1.569593000      -4.568655000       2.014723000
H         0.141641000      -4.093769000       2.206671000
H        -1.107459000      -3.258036000       3.136520000
H         4.539174000      -0.325941000      -0.002740000
```

H	-2.554308000	-3.765782000	0.000141000
H	-1.987055000	4.092686000	-0.000306000

[Fe(tfaa)3] *fac*

43
symmetry c1

Fe	0.000626000	0.004860000	0.722227000
O	-1.634563000	-0.193477000	1.884398000
O	-1.176454000	1.153509000	-0.443313000
O	0.646734000	1.530466000	1.871544000
O	1.585389000	0.439402000	-0.445383000
O	1.000147000	-1.312288000	1.876663000
O	-0.417736000	-1.590960000	-0.436883000
C	1.239371000	-2.594616000	1.795070000
C	0.739454000	-3.397276000	0.709289000
C	-0.040283000	-2.841206000	-0.305659000
C	-2.865071000	0.239726000	1.804027000
C	-3.314257000	1.065804000	0.713809000
C	-2.447233000	1.453364000	-0.308912000
C	1.640832000	2.374775000	1.787621000
C	2.583024000	2.339388000	0.699371000
C	2.484550000	1.386799000	-0.315546000
C	-2.974527000	2.350907000	-1.451497000
F	-4.334473000	2.677599000	-1.328763000
F	-2.270549000	3.569901000	-1.516916000
F	-2.818161000	1.734990000	-2.708319000
C	-3.813786000	-0.160900000	2.914517000
H	-3.420472000	0.194632000	3.875398000
H	-4.816402000	0.243273000	2.760861000
H	-3.866607000	-1.255545000	2.971170000
C	-0.565079000	-3.747081000	-1.442762000
F	-1.973434000	-3.748162000	-1.493168000
F	-0.123560000	-3.303186000	-2.704119000
F	-0.165456000	-5.087866000	-1.324333000
C	2.062348000	-3.217608000	2.903396000
H	2.987196000	-2.644877000	3.040196000
H	1.501604000	-3.159347000	3.846051000
H	2.302887000	-4.262337000	2.695642000
C	3.528969000	1.380652000	-1.454876000
F	2.922082000	1.542533000	-2.715125000
F	4.494622000	2.393294000	-1.339171000
F	4.229325000	0.158738000	-1.505843000
C	1.777241000	3.397317000	2.896544000
H	2.542651000	4.143344000	2.672569000
H	0.813199000	3.892019000	3.061827000
H	2.043135000	2.882660000	3.830035000
H	-4.344935000	1.393146000	0.697904000
H	3.383966000	3.065832000	0.678846000
H	0.972458000	-4.453207000	0.690836000

[Fe(tfaa)3] mer

```
43
symmetry c1
Fe        0.052568000    -0.335128000    -0.155191000
O         0.551030000    -2.132852000    -0.920728000
O         2.020388000    -0.161133000     0.254755000
O         0.185409000     0.514877000    -1.978209000
O        -0.242393000     1.541352000     0.515628000
O        -1.899616000    -0.714902000    -0.498805000
O        -0.308775000    -1.046753000     1.693620000
C        -2.804847000    -1.218625000     0.306537000
C        -2.642707000    -1.618068000     1.634225000
C        -1.370051000    -1.512790000     2.298407000
C         1.670385000    -2.799151000    -1.028674000
C         2.921826000    -2.271531000    -0.551100000
C         2.997747000    -1.011352000     0.043911000
C         0.116198000     1.745024000    -2.412858000
C        -0.112034000     2.857188000    -1.526225000
C        -0.268238000     2.671443000    -0.152514000
C         4.365910000    -0.483999000     0.532641000
F         5.421901000    -1.387450000     0.332581000
F         4.724271000     0.709047000    -0.126275000
F         4.344390000    -0.185604000     1.908857000
C         1.604087000    -4.162173000    -1.685588000
H         1.242810000    -4.050992000    -2.716223000
H         2.576480000    -4.658802000    -1.695744000
H         0.877419000    -4.789016000    -1.154030000
C        -1.210148000    -1.945157000     3.740947000
H        -0.442409000    -2.726743000     3.804280000
H        -0.856316000    -1.095100000     4.338035000
H        -2.145582000    -2.319842000     4.161427000
C        -4.190698000    -1.357739000    -0.363774000
F        -4.670427000    -0.115582000    -0.823431000
F        -4.136680000    -2.202922000    -1.489982000
F        -5.177757000    -1.881725000     0.486012000
C        -0.513071000     3.896908000     0.756456000
F         0.481447000     4.013518000     1.747130000
F        -0.536061000     5.117372000     0.062839000
F        -1.742028000     3.796738000     1.438730000
C         0.310175000     1.970628000    -3.897814000
H        -0.076074000     2.942228000    -4.215075000
H         1.385127000     1.933734000    -4.127152000
H        -0.175268000     1.168603000    -4.462641000
H         3.816733000    -2.868260000    -0.663363000
H        -0.160902000     3.854078000    -1.942996000
H        -3.485828000    -2.015408000     2.182732000
```

[Fe(ba)$_3$] *fac*

```
64
```

symmetry c1

Fe	-0.013812000	-0.006303000	1.270368000
O	0.324684000	1.609010000	0.110645000
O	-1.081906000	1.253838000	2.423777000
O	-1.575035000	-0.529353000	0.107085000
O	-0.575233000	-1.551918000	2.434300000
O	1.217675000	-1.108426000	0.114872000
O	1.610234000	0.295544000	2.423904000
C	2.502366000	-1.409459000	0.193921000
C	3.326208000	-0.943277000	1.256718000
C	2.857752000	-0.123190000	2.314817000
C	-0.022545000	2.880617000	0.208898000
C	-0.817721000	3.368543000	1.283927000
C	-1.311536000	2.550919000	2.332306000
C	-2.492888000	-1.476050000	0.199323000
C	-2.523158000	-2.399817000	1.281769000
C	-1.580157000	-2.403348000	2.341252000
C	-2.163521000	3.149794000	3.441528000
H	-2.316754000	4.223583000	3.305511000
H	-3.138827000	2.646624000	3.468379000
H	-1.680627000	2.973999000	4.411605000
C	3.804938000	0.331379000	3.415250000
H	3.829005000	1.428317000	3.449975000
H	3.432467000	-0.016257000	4.387647000
H	4.819652000	-0.045855000	3.263352000
C	-1.682327000	-3.427938000	3.461364000
H	-1.786616000	-2.910706000	4.424048000
H	-2.530739000	-4.103160000	3.322252000
H	-0.756602000	-4.016143000	3.507394000
H	-1.078456000	4.417728000	1.319412000
H	-3.301375000	-3.150143000	1.318732000
H	4.372031000	-1.218323000	1.278864000
C	-3.509705000	-1.521079000	-0.909182000
C	-3.438474000	-0.539303000	-1.936472000
C	-4.535353000	-2.506079000	-0.970708000
C	-4.367806000	-0.539263000	-2.997636000
C	-5.463933000	-2.505633000	-2.033617000
C	-5.384852000	-1.523049000	-3.050243000
H	-2.655010000	0.209441000	-1.886928000
H	-4.618385000	-3.273445000	-0.207665000
H	-4.299958000	0.218664000	-3.774213000
H	-6.240841000	-3.265370000	-2.067494000
H	-6.101993000	-1.525027000	-3.867739000
C	0.472742000	3.787900000	-0.884890000
C	0.205718000	5.185885000	-0.902023000
C	1.239366000	3.222645000	-1.941797000
C	0.694244000	5.994242000	-1.950746000
C	1.726029000	4.031409000	-2.990068000
C	1.455264000	5.421249000	-2.998484000
H	-0.373027000	5.652945000	-0.111584000
H	1.441230000	2.156863000	-1.925578000

```
H          0.482674000       7.060607000      -1.949230000
H          2.309634000       3.583585000      -3.790672000
H          1.829969000       6.046556000      -3.805426000
C          3.046664000      -2.279084000      -0.907042000
C          2.160293000      -2.710251000      -1.933049000
C          4.409663000      -2.685605000      -0.961450000
C          2.622126000      -3.526101000      -2.987047000
C          4.870532000      -3.501816000      -2.016606000
C          3.979935000      -3.925226000      -3.032887000
H          1.122130000      -2.398604000      -1.888018000
H          5.115129000      -2.376385000      -0.196775000
H          1.931820000      -3.847012000      -3.763335000
H          5.914520000      -3.803830000      -2.044431000
H          4.337963000      -4.554107000      -3.844612000
```

[Fe(ba)$_3$] *mer*

```
64
symmetry c1
Fe         0.018945000      -0.626294000       0.145700000
O         -0.017685000       1.374817000      -0.104479000
O          0.018137000      -0.209893000       2.117813000
O         -1.989421000      -0.683117000      -0.012060000
O         -0.124041000      -2.596038000       0.544657000
O          0.190785000      -0.879303000      -1.846636000
O          2.029811000      -0.761093000       0.167011000
C          1.242575000      -1.057936000      -2.624240000
C          2.577802000      -1.106326000      -2.147770000
C          2.933228000      -0.960861000      -0.777377000
C         -0.044666000       2.391698000       0.739573000
C         -0.037166000       2.205908000       2.150643000
C         -0.005516000       0.933863000       2.777103000
C         -2.878230000      -1.655710000       0.094983000
C         -2.503452000      -2.994233000       0.400686000
C         -1.163337000      -3.407547000       0.613088000
C          0.000412000       0.821614000       4.294425000
H         -0.017431000       1.802265000       4.777255000
H         -0.871380000       0.241368000       4.623758000
H          0.895519000       0.275410000       4.619195000
C         -0.846615000      -4.859152000       0.940554000
H         -0.329210000      -4.913245000       1.907229000
H         -1.748391000      -5.475833000       0.980525000
H         -0.163780000      -5.266559000       0.183690000
H         -0.060601000       3.069863000       2.800969000
H         -3.267300000      -3.755898000       0.479698000
H          3.353829000      -1.261171000      -2.885218000
C         -4.314957000      -1.265489000      -0.124455000
C         -4.610040000       0.089268000      -0.443753000
C         -5.386937000      -2.196712000      -0.025597000
C         -5.940776000       0.504239000      -0.659988000
C         -6.717988000      -1.780130000      -0.241656000
```

C	-7.000558000	-0.429470000	-0.559628000
H	-3.790792000	0.796494000	-0.518721000
H	-5.200895000	-3.238021000	0.217429000
H	-6.149118000	1.543125000	-0.903803000
H	-7.525880000	-2.503275000	-0.162353000
H	-8.027131000	-0.111474000	-0.725816000
C	-0.081803000	3.763569000	0.122090000
C	-0.075379000	4.955303000	0.900411000
C	-0.124753000	3.870284000	-1.296045000
C	-0.110342000	6.219102000	0.273109000
C	-0.161086000	5.134038000	-1.921766000
C	-0.153753000	6.314053000	-1.139094000
H	-0.040475000	4.913332000	1.984406000
H	-0.130146000	2.960344000	-1.886793000
H	-0.103576000	7.120145000	0.881355000
H	-0.194558000	5.198208000	-3.006612000
H	-0.181053000	7.289216000	-1.619598000
C	0.946585000	-1.217497000	-4.108113000
H	1.857697000	-1.364655000	-4.693944000
H	0.277525000	-2.074529000	-4.259518000
H	0.421091000	-0.326584000	-4.475898000
C	4.365416000	-1.023901000	-0.319574000
C	5.448335000	-1.247989000	-1.215717000
C	4.644620000	-0.853834000	1.065125000
C	6.774579000	-1.299464000	-0.735824000
C	5.970648000	-0.904897000	1.543511000
C	7.041437000	-1.127892000	0.644247000
H	5.273976000	-1.384438000	-2.278190000
H	3.816953000	-0.684159000	1.745590000
H	7.590872000	-1.472134000	-1.432786000
H	6.166927000	-0.772667000	2.604696000
H	8.064368000	-1.167845000	1.011119000

[Fe(tfba)$_3$] *fac*

64
symmetry c1

Fe	0.011164000	0.000911000	-0.497652000
O	-0.374822000	1.612303000	0.646207000
O	1.032451000	1.289080000	-1.658636000
O	1.584790000	-0.472976000	0.663869000
O	0.628827000	-1.523882000	-1.656194000
O	-1.189426000	-1.133374000	0.653580000
O	-1.617083000	0.237527000	-1.655681000
C	-2.440440000	-1.523691000	0.590459000
C	-3.289881000	-1.108947000	-0.500889000
C	-2.818847000	-0.278113000	-1.513595000
C	-0.108246000	2.894411000	0.565479000
C	0.668757000	3.420233000	-0.531957000
C	1.168266000	2.591312000	-1.532363000
C	2.550528000	-1.359093000	0.603858000

C	2.631350000	-2.290953000	-0.496030000
C	1.683730000	-2.296988000	-1.515726000
C	2.009409000	3.208499000	-2.670830000
F	2.164689000	4.601422000	-2.571259000
F	3.308572000	2.661525000	-2.709288000
F	1.438491000	2.957012000	-3.934242000
C	-3.778071000	0.143819000	-2.647710000
F	-3.934964000	1.544374000	-2.698022000
F	-3.291243000	-0.243493000	-3.911993000
F	-5.068747000	-0.399157000	-2.531605000
C	1.820353000	-3.321252000	-2.663257000
F	1.946923000	-2.685432000	-3.914741000
F	2.929109000	-4.174554000	-2.533416000
F	0.684184000	-4.150700000	-2.756094000
H	0.883823000	4.476916000	-0.585559000
H	3.439200000	-3.005914000	-0.541653000
H	-4.316900000	-1.439427000	-0.543419000
C	3.549411000	-1.359267000	1.710752000
C	3.422104000	-0.382240000	2.740269000
C	4.618657000	-2.299077000	1.773205000
C	4.342561000	-0.343929000	3.805321000
C	5.537192000	-2.258134000	2.842037000
C	5.403464000	-1.281936000	3.859252000
H	2.606077000	0.330671000	2.688053000
H	4.743452000	-3.059908000	1.009804000
H	4.236857000	0.406531000	4.584058000
H	6.348354000	-2.979974000	2.880900000
H	6.114460000	-1.252892000	4.681212000
C	-0.635183000	3.773694000	1.648122000
C	-0.432637000	5.184172000	1.650583000
C	-1.366742000	3.174878000	2.714351000
C	-0.951420000	5.973311000	2.697391000
C	-1.881750000	3.966577000	3.758946000
C	-1.675555000	5.368645000	3.753577000
H	0.116402000	5.674421000	0.853396000
H	-1.518478000	2.100717000	2.707341000
H	-0.792484000	7.048107000	2.689111000
H	-2.437176000	3.499391000	4.567680000
H	-2.073285000	5.980452000	4.559477000
C	-2.939203000	-2.404482000	1.685146000
C	-2.036893000	-2.773806000	2.724447000
C	-4.279935000	-2.885233000	1.725667000
C	-2.464438000	-3.603453000	3.778971000
C	-4.703977000	-3.715430000	2.783359000
C	-3.799780000	-4.076869000	3.811623000
H	-1.017124000	-2.405512000	2.688537000
H	-4.994120000	-2.624079000	0.951737000
H	-1.767879000	-3.879122000	4.566151000
H	-5.728560000	-4.076395000	2.804982000
H	-4.130447000	-4.717361000	4.625519000

[Fe(tfba)$_3$] *mer*

```
64
symmetry c1
Fe      -0.049526000    -0.231883000     0.070311000
O        0.247674000     1.706871000    -0.386504000
O       -0.143137000     0.418462000     1.975105000
O       -2.021162000    -0.044742000    -0.269011000
O       -0.505636000    -2.090626000     0.692687000
O        0.223838000    -0.744704000    -1.858507000
O        1.906777000    -0.647551000     0.276720000
C        1.322260000    -1.144105000    -2.461337000
C        2.591389000    -1.324520000    -1.918381000
C        2.858687000    -1.067044000    -0.523341000
C        0.353852000     2.820064000     0.299912000
C        0.239412000     2.814685000     1.739033000
C        0.004877000     1.637986000     2.444721000
C       -3.069447000    -0.817247000    -0.106573000
C       -2.928717000    -2.151053000     0.427473000
C       -1.684155000    -2.668444000     0.776021000
C       -0.107570000     1.694025000     3.983772000
F        0.014083000     2.988894000     4.515438000
F       -1.344106000     1.189581000     4.433851000
F        0.886873000     0.909965000     4.604163000
C       -1.597215000    -4.106038000     1.333522000
F       -1.017954000    -4.131008000     2.618084000
F       -2.841720000    -4.748955000     1.442424000
F       -0.789859000    -4.928985000     0.521714000
H        0.328177000     3.737182000     2.292895000
H       -3.799361000    -2.775819000     0.559454000
H        3.383255000    -1.668760000    -2.566507000
C       -4.396903000    -0.260792000    -0.494429000
C       -4.450352000     1.058508000    -1.030421000
C       -5.605827000    -1.000595000    -0.347090000
C       -5.682512000     1.624507000    -1.410575000
C       -6.837330000    -0.429857000    -0.728163000
C       -6.880071000     0.881804000    -1.260421000
H       -3.527043000     1.617264000    -1.140781000
H       -5.602843000    -2.006720000     0.059043000
H       -5.711725000     2.631129000    -1.818807000
H       -7.753450000    -1.002166000    -0.611067000
H       -7.831651000     1.318422000    -1.553464000
C        0.594934000     4.076744000    -0.465162000
C        0.760577000     5.338804000     0.175452000
C        0.661209000     4.004986000    -1.886880000
C        0.987261000     6.499446000    -0.592114000
C        0.885985000     5.167131000    -2.649881000
C        1.049854000     6.418150000    -2.004589000
H        0.719551000     5.430298000     1.255879000
H        0.534367000     3.041920000    -2.369891000
H        1.113611000     7.456681000    -0.093823000
```

H	0.933329000	5.101254000	-3.733484000
H	1.223945000	7.315462000	-2.593233000
C	1.098441000	-1.432219000	-3.961732000
F	2.249726000	-1.876687000	-4.633079000
F	0.111590000	-2.418903000	-4.159745000
F	0.650647000	-0.284772000	-4.648419000
C	4.213489000	-1.261212000	0.067448000
C	5.331268000	-1.681209000	-0.710337000
C	4.388870000	-1.015274000	1.460167000
C	6.593293000	-1.849325000	-0.104797000
C	5.650934000	-1.185921000	2.061184000
C	6.757319000	-1.603330000	1.280211000
H	5.235120000	-1.875339000	-1.773618000
H	3.534697000	-0.696874000	2.048274000
H	7.439479000	-2.168875000	-0.706795000
H	5.773027000	-0.997006000	3.124281000
H	7.731956000	-1.734865000	1.743616000

[Fe(tffu)$_3$] *fac*

55
symmetry c1

Fe	-0.001514000	0.002881000	-0.253944000
O	-1.645493000	0.060238000	0.919511000
O	-0.965555000	1.340772000	-1.399124000
O	0.877531000	1.400794000	0.909595000
O	1.635015000	0.161004000	-1.406824000
O	0.775624000	-1.451608000	0.912612000
O	-0.686618000	-1.498903000	-1.397455000
C	0.785719000	-2.759410000	0.835578000
C	0.142730000	-3.476685000	-0.242859000
C	-0.529017000	-2.799657000	-1.253652000
C	-2.785129000	0.701873000	0.842517000
C	-3.090476000	1.610761000	-0.239869000
C	-2.171627000	1.852537000	-1.253960000
C	2.006558000	2.060357000	0.828402000
C	2.943443000	1.861856000	-0.254889000
C	2.685770000	0.944416000	-1.266379000
C	-2.536711000	2.837981000	-2.382721000
F	-3.817917000	3.400348000	-2.251165000
F	-1.629687000	3.915948000	-2.444552000
F	-2.497436000	2.218618000	-3.648162000
C	-1.206272000	-3.606242000	-2.380320000
F	-2.594069000	-3.361320000	-2.432403000
F	-0.698444000	-3.258353000	-3.648198000
F	-1.050062000	-4.997123000	-2.253792000
C	3.717817000	0.759399000	-2.397410000
F	3.157836000	1.026384000	-3.662853000
F	4.846358000	1.587998000	-2.275532000
F	4.196937000	-0.565874000	-2.451482000
H	-4.046720000	2.114940000	-0.260852000

H	3.860984000	2.433360000	-0.279157000
H	0.181885000	-4.557049000	-0.264468000
C	1.472113000	-3.511639000	1.877462000
C	1.673338000	-4.874815000	2.111666000
C	2.441490000	-4.993175000	3.323113000
C	2.676138000	-3.702181000	3.775596000
O	2.096367000	-2.774654000	2.913873000
H	1.318740000	-5.691503000	1.499273000
H	2.773599000	-5.906936000	3.793354000
H	3.197385000	-3.292758000	4.627326000
C	-3.775765000	0.487886000	1.889175000
C	-5.058509000	0.991492000	2.123058000
C	-5.538190000	0.394409000	3.341699000
C	-4.531915000	-0.445141000	3.798856000
O	-3.441977000	-0.409939000	2.932966000
H	-5.594358000	1.698649000	1.506165000
H	-6.494825000	0.563509000	3.813530000
H	-4.431885000	-1.092553000	4.656544000
C	2.323117000	3.027382000	1.870938000
C	3.406260000	3.880370000	2.100751000
C	3.133838000	4.601551000	3.316248000
C	1.900689000	4.160064000	3.775465000
O	1.379889000	3.197891000	2.914036000
H	4.287148000	3.982111000	1.483127000
H	3.763522000	5.343483000	3.784648000
H	1.291530000	4.405084000	4.632013000

[Fe(tffu)$_3$] *mer*

55
symmetry c1

Fe	0.037814000	-0.105503000	-0.073410000
O	-0.528038000	1.638582000	0.774350000
O	0.230716000	0.966271000	-1.764060000
O	1.940269000	0.204614000	0.516933000
O	0.752933000	-1.730386000	-1.013687000
O	-0.327118000	-1.076023000	1.651377000
O	-1.842341000	-0.625979000	-0.581269000
C	-1.413719000	-1.713382000	2.036413000
C	-2.605902000	-1.876551000	1.340050000
C	-2.782841000	-1.312151000	0.021488000
C	-0.669424000	2.859390000	0.319618000
C	-0.408547000	3.207713000	-1.058798000
C	0.012185000	2.248878000	-1.972704000
C	3.061147000	-0.444581000	0.317353000
C	3.126703000	-1.653063000	-0.473120000
C	1.987906000	-2.187513000	-1.064342000
C	0.279965000	2.665855000	-3.433165000
F	0.060251000	4.031439000	-3.681984000
F	1.610517000	2.397411000	-3.814666000
F	-0.538224000	1.956316000	-4.335468000

C	2.107058000	-3.481476000	-1.895221000
F	1.670783000	-3.291125000	-3.221713000
F	3.416183000	-3.986493000	-1.972328000
F	1.308674000	-4.515214000	-1.363502000
H	-0.542224000	4.229702000	-1.385670000
H	4.076709000	-2.151731000	-0.607476000
H	-3.413006000	-2.434278000	1.794627000
C	-1.106821000	3.899454000	1.241096000
C	-1.348335000	5.269401000	1.105142000
C	-1.764020000	5.761422000	2.392288000
C	-1.759707000	4.675045000	3.256213000
O	-1.362611000	3.522859000	2.582541000
H	-1.241984000	5.854260000	0.202634000
H	-2.028990000	6.777967000	2.642933000
H	-1.993379000	4.552323000	4.302678000
C	4.282089000	0.077531000	0.915494000
C	5.615324000	-0.341974000	0.911047000
C	6.365141000	0.604021000	1.694840000
C	5.461504000	1.557691000	2.142720000
O	4.180372000	1.263800000	1.683043000
H	6.012838000	-1.214717000	0.412926000
H	7.425502000	0.580945000	1.898449000
H	5.552044000	2.444301000	2.751403000
C	-1.272489000	-2.329497000	3.443491000
F	-2.411092000	-3.033644000	3.870730000
F	-0.192705000	-3.233226000	3.509999000
F	-1.019927000	-1.343887000	4.419673000
C	-4.051613000	-1.505701000	-0.667419000
C	-5.238789000	-2.164993000	-0.336067000
C	-6.143829000	-2.000991000	-1.443076000
C	-5.474074000	-1.250312000	-2.399414000
O	-4.193352000	-0.932304000	-1.954971000
H	-5.442083000	-2.701140000	0.579978000
H	-7.150212000	-2.385219000	-1.519968000
H	-5.738610000	-0.880290000	-3.378141000

[Fe(tta)$_3$] *fac*

55
symmetry c1

C	2.642332000	-2.741245000	1.650877000
C	3.302967000	-3.962779000	1.816603000
C	4.138962000	-4.015487000	2.984845000
C	4.126410000	-2.838796000	3.721957000
C	0.485967000	-2.813955000	-1.477739000
C	1.365978000	-3.206479000	-0.476188000
C	1.721378000	-2.310805000	0.602865000
C	0.458680000	5.003924000	3.699279000
C	1.464287000	5.596422000	2.947129000
C	1.817830000	4.841143000	1.776200000
C	1.083034000	3.661009000	1.623581000

```
C          1.151430000       2.645509000       0.576636000
C          2.090180000       2.778971000      -0.515900000
C          2.171430000       1.818797000      -1.517500000
C          3.196589000       2.003281000      -2.655329000
C         -4.519199000      -2.074450000       3.769909000
C         -5.544601000      -1.502694000       3.028489000
C         -5.082840000      -0.827570000       1.846306000
C         -3.695813000      -0.878048000       1.674048000
C         -2.863712000      -0.320026000       0.612021000
C         -3.461976000       0.414632000      -0.481152000
C         -2.682669000       0.958854000      -1.495233000
C         -3.368312000       1.742383000      -2.633495000
Fe        -0.004251000      -0.003728000      -0.481811000
F          3.965296000       3.174251000      -2.541895000
F          2.570731000       2.054224000      -3.917193000
F          4.108301000       0.929001000      -2.711804000
F         -4.767425000       1.804213000      -2.516948000
F         -3.094825000       1.175823000      -3.894610000
F         -2.911469000       3.075268000      -2.694372000
O         -0.127443000      -1.655965000      -1.617405000
O          1.235655000      -1.091992000       0.681592000
O          0.334898000       1.619651000       0.668686000
O          1.469782000       0.710638000      -1.644918000
O         -1.565725000      -0.511431000       0.691205000
O         -1.373155000       0.909345000      -1.634130000
S          3.063212000      -1.581996000       2.998374000
S         -0.114486000       3.455189000       2.987548000
S         -2.901240000      -1.804818000       3.033127000
H          2.584889000       5.163720000       1.080335000
H          1.933904000       6.535234000       3.218413000
H          0.007332000       5.356089000       4.617796000
H          2.745181000       3.637817000      -0.556074000
H         -4.534113000       0.547588000      -0.512548000
H         -5.755182000      -0.327576000       1.157246000
H         -6.588543000      -1.561153000       3.315273000
H         -4.586599000      -2.634928000       4.693341000
H          3.200044000      -4.797730000       1.131771000
H          4.722058000      -4.885496000       3.265123000
H          1.785969000      -4.201948000      -0.505226000
H          4.659469000      -2.610029000       4.635733000
C          0.127876000      -3.810906000      -2.599065000
F         -1.255591000      -4.083344000      -2.628776000
F          0.455395000      -3.303140000      -3.872241000
F          0.777033000      -5.051934000      -2.483714000
```

[Fe(tta)$_3$] *mer*

```
55
symmetry c1
C         -6.241207000      -1.368768000      -1.453326000
C         -6.518982000      -1.698836000      -0.133427000
```

C	-5.369827000	-1.617738000	0.726315000
C	-4.200265000	-1.223987000	0.068132000
C	-2.851983000	-1.030975000	0.593289000
C	-2.569693000	-1.252963000	1.994112000
C	-1.290177000	-1.075266000	2.507902000
C	-1.041512000	-1.322690000	4.010060000
C	6.311362000	1.161557000	1.150274000
C	6.719484000	-0.109238000	0.767335000
C	5.632916000	-0.953732000	0.352052000
C	4.380958000	-0.333212000	0.414534000
C	3.059902000	-0.857313000	0.080805000
C	2.906488000	-2.213479000	-0.397769000
C	1.655158000	-2.730200000	-0.712948000
C	1.546877000	-4.184383000	-1.216232000
C	-0.934815000	5.841017000	2.077738000
C	-0.916876000	6.350307000	0.786109000
C	-0.707906000	5.343713000	-0.218700000
C	-0.563854000	4.052232000	0.298561000
C	-0.340239000	2.786015000	-0.393327000
C	-0.239669000	2.743011000	-1.835583000
C	-0.017862000	1.545465000	-2.505544000
C	0.085670000	1.552553000	-4.044666000
Fe	0.049136000	-0.253934000	-0.074793000
F	-2.190088000	-1.714922000	4.718446000
F	-0.548066000	-0.168863000	4.652770000
F	-0.077447000	-2.330320000	4.218651000
F	2.782548000	-4.847923000	-1.303656000
F	0.964462000	-4.249251000	-2.498372000
F	0.730083000	-4.966564000	-0.373921000
F	-0.052122000	2.828947000	-4.616548000
F	-0.901915000	0.738717000	-4.636350000
F	1.326168000	1.047408000	-4.485093000
O	-1.918992000	-0.649300000	-0.251175000
O	-0.197580000	-0.710201000	1.869097000
O	2.030126000	-0.053800000	0.231214000
O	0.482905000	-2.133333000	-0.638139000
O	-0.236106000	1.701090000	0.341792000
O	0.128081000	0.339668000	-1.995199000
S	-4.515158000	-0.933280000	-1.707699000
S	4.532222000	1.385238000	1.015815000
S	-0.689510000	4.060159000	2.121276000
H	5.778996000	-1.976833000	0.022310000
H	7.755182000	-0.429508000	0.781342000
H	6.917241000	1.986496000	1.502089000
H	3.778693000	-2.841422000	-0.512012000
H	-0.335331000	3.655727000	-2.406428000
H	-0.666374000	5.573860000	-1.277952000
H	-1.047602000	7.402774000	0.560957000
H	-1.072772000	6.372942000	3.010184000
H	-6.916132000	-1.347792000	-2.299151000
H	-7.505290000	-1.988603000	0.211147000

```
H        -5.413687000      -1.842859000       1.786557000
H        -3.365084000      -1.562977000       2.657066000
```

[Fe(dbm)$_3$]

```
85
symmetry c1
Fe        0.000871000      -0.004661000       0.000161000
O         0.751432000       1.477698000      -1.135037000
O        -0.738440000       1.479098000       1.138842000
O        -1.614460000      -0.121175000      -1.194352000
O        -0.958386000      -1.364636000       1.130517000
O         0.952553000      -1.364708000      -1.134402000
O         1.615474000      -0.133276000       1.194561000
C         2.121435000      -1.973167000      -1.022887000
C         3.004145000      -1.744778000       0.066721000
C         2.728014000      -0.845308000       1.131680000
C         0.694672000       2.796085000      -1.046492000
C         0.011327000       3.465769000       0.003945000
C        -0.675287000       2.797325000       1.052966000
C        -2.731846000      -0.825814000      -1.132957000
C        -3.014118000      -1.725336000      -0.069722000
C        -2.133245000      -1.961998000       1.019735000
H         0.013896000       4.544704000       0.005171000
H        -3.951601000      -2.259170000      -0.091119000
H         3.939082000      -2.283402000       0.085166000
C         3.699784000      -0.644161000       2.264272000
C         4.936237000      -1.343845000       2.352571000
C         3.364387000       0.285309000       3.287957000
C         5.811075000      -1.117562000       3.436517000
C         4.239772000       0.511093000       4.370753000
C         5.467477000      -0.189751000       4.449465000
H         5.226783000      -2.062705000       1.593153000
H         2.421414000       0.817283000       3.219110000
H         6.751338000      -1.660789000       3.489022000
H         3.967910000       1.225318000       5.144171000
H         6.143784000      -0.017011000       5.283355000
C         1.404638000       3.558043000      -2.134334000
C         1.458299000       4.980100000      -2.171167000
C         2.047276000       2.821799000      -3.168354000
C         2.137590000       5.644058000      -3.214823000
C         2.725157000       3.486452000      -4.211634000
C         2.773162000       4.901215000      -4.239161000
H         0.982164000       5.577789000      -1.400346000
H         2.004263000       1.738228000      -3.139143000
H         2.169650000       6.730682000      -3.227482000
H         3.210136000       2.907988000      -4.994165000
H         3.295459000       5.415535000      -5.042432000
C        -1.382935000       3.560668000       2.141246000
C        -2.030721000       2.825456000       3.172763000
C        -1.429806000       4.982967000       2.180816000
```

C	-2.706769000	3.491185000	4.216517000
C	-2.107295000	5.647968000	3.224965000
C	-2.748097000	4.906098000	4.246778000
H	-1.993009000	1.741815000	3.141223000
H	-0.949819000	5.580194000	1.411950000
H	-3.195658000	2.913425000	4.997134000
H	-2.134155000	6.734689000	3.239841000
H	-3.268801000	5.421331000	5.050492000
C	-3.701894000	-0.617775000	-2.266036000
C	-3.357631000	0.306158000	-3.291848000
C	-4.944414000	-1.306922000	-2.353589000
C	-4.230077000	0.536828000	-4.375973000
C	-5.816319000	-1.075622000	-3.438879000
C	-5.463737000	-0.153522000	-4.453972000
H	-2.410030000	0.829923000	-3.223629000
H	-5.242534000	-2.020938000	-1.592428000
H	-3.951246000	1.246469000	-5.151123000
H	-6.761336000	-1.610594000	-3.490763000
H	-6.137716000	0.022885000	-5.288969000
C	-2.487714000	-2.922129000	2.124527000
C	-1.551590000	-3.117449000	3.177922000
C	-3.717153000	-3.638875000	2.159282000
C	-1.833756000	-4.004820000	4.237438000
C	-3.998565000	-4.526416000	3.219812000
C	-3.059056000	-4.713754000	4.262552000
H	-0.615815000	-2.569555000	3.149887000
H	-4.458150000	-3.516164000	1.375834000
H	-1.106520000	-4.142205000	5.033946000
H	-4.942409000	-5.065813000	3.231325000
H	-3.278502000	-5.398513000	5.078344000
C	2.467843000	-2.935736000	-2.128186000
C	1.537344000	-3.110238000	-3.190255000
C	3.684199000	-3.674815000	-2.154925000
C	1.812830000	-3.998039000	-4.251091000
C	3.958483000	-4.563525000	-3.216408000
C	3.025159000	-4.729198000	-4.268329000
H	0.611534000	-2.545422000	-3.168297000
H	4.420200000	-3.569405000	-1.364091000
H	1.090501000	-4.118582000	-5.054751000
H	4.892124000	-5.120467000	-3.221623000
H	3.239354000	-5.414480000	-5.085054000

[Fe(bth)$_3$] *fac*

76
symmetry c1

Fe	-0.016289000	0.005777000	-0.046737000
O	1.262114000	-0.974711000	1.151648000
O	-0.073474000	-1.708839000	-1.109319000
O	-1.557267000	-0.496321000	1.137077000
O	-1.422624000	0.802727000	-1.254296000

O	0.274018000	1.698648000	0.991489000
O	1.428833000	0.704675000	-1.270258000
C	1.090944000	2.729802000	0.837802000
C	2.016914000	2.815226000	-0.233499000
C	2.152814000	1.812193000	-1.234199000
C	1.778065000	-2.194212000	1.125598000
C	1.453031000	-3.137440000	0.117055000
C	0.548555000	-2.866076000	-0.947786000
C	-2.868381000	-0.335632000	1.040523000
C	-3.476122000	0.319812000	-0.060983000
C	-2.741274000	0.860111000	-1.153575000
H	1.906928000	-4.117388000	0.152396000
H	-4.551417000	0.424221000	-0.078165000
H	2.662189000	3.679523000	-0.298097000
C	-3.684544000	-0.894025000	2.175096000
C	-3.002632000	-1.506017000	3.263529000
C	-5.106607000	-0.836520000	2.201847000
C	-3.720824000	-2.045006000	4.351445000
C	-5.823985000	-1.377343000	3.290158000
C	-5.135231000	-1.982812000	4.369200000
H	-1.918883000	-1.549080000	3.239845000
H	-5.663102000	-0.381783000	1.388348000
H	-3.183910000	-2.508459000	5.175523000
H	-6.909955000	-1.326650000	3.294811000
H	-5.690673000	-2.398348000	5.206680000
C	2.738857000	-2.525571000	2.235856000
C	3.402764000	-3.781189000	2.330863000
C	2.992916000	-1.539192000	3.229207000
C	4.296499000	-4.039291000	3.392103000
C	3.885490000	-1.798888000	4.290199000
C	4.541573000	-3.050634000	4.375848000
H	3.237533000	-4.558471000	1.591428000
H	2.485380000	-0.583344000	3.154623000
H	4.796541000	-5.002922000	3.449576000
H	4.067555000	-1.033928000	5.041105000
H	5.230608000	-3.252519000	5.192562000
C	0.979986000	3.816509000	1.873119000
C	0.076728000	3.630836000	2.956564000
C	1.738513000	5.019424000	1.810975000
C	-0.063838000	4.618426000	3.953956000
C	1.595203000	6.007719000	2.808150000
C	0.695187000	5.811585000	3.883699000
H	-0.500682000	2.713473000	3.000709000
H	2.432537000	5.201082000	0.996375000
H	-0.757509000	4.459661000	4.776017000
H	2.180143000	6.921939000	2.745028000
H	0.587387000	6.574544000	4.651059000
C	0.252290000	-3.904978000	-1.954538000
C	0.715161000	-5.214013000	-2.069159000
C	0.185658000	-5.925921000	-3.207348000
C	-0.686484000	-5.168920000	-3.972562000

```
S        -0.902652000      -3.504305000      -3.312882000
H         1.410386000      -5.664643000      -1.368634000
H         0.444048000      -6.953217000      -3.441521000
H        -1.220729000      -5.451295000      -4.870444000
C         3.147377000       1.969073000      -2.314394000
C         4.055306000       2.994676000      -2.568915000
C         4.872022000       2.778117000      -3.738869000
C         4.597089000       1.585927000      -4.388711000
S         3.288417000       0.651034000      -3.572517000
H         4.146208000       3.879525000      -1.947661000
H         5.627539000       3.479295000      -4.077208000
H         5.055132000       1.178659000      -5.280716000
C        -3.444668000       1.535720000      -2.262230000
C        -4.803243000       1.765958000      -2.467895000
C        -5.099915000       2.471264000      -3.691495000
C        -3.972276000       2.786731000      -4.431869000
S        -2.455544000       2.216773000      -3.639787000
H        -5.574919000       1.446179000      -1.775492000
H        -6.106783000       2.729451000      -4.002145000
H        -3.906673000       3.307034000      -5.378613000
```

[Fe(bth)$_3$] *mer*

```
76
symmetry c1
C        -2.329956000      -2.303906000       5.636816000
C        -3.603480000      -1.830285000       5.366656000
C        -3.700920000      -1.134809000       4.105768000
C        -2.501385000      -1.070788000       3.400178000
C        -2.185525000      -0.457919000       2.094165000
C        -3.199365000       0.207349000       1.349627000
C        -2.967761000       0.826803000       0.094311000
C         2.524927000       1.995039000      -5.686273000
C         3.029354000       3.156045000      -5.123462000
C         2.740220000       3.282750000      -3.715259000
C         2.010634000       2.218947000      -3.188973000
C         1.523564000       1.973469000      -1.816697000
C         1.793239000       2.911782000      -0.781310000
C         1.356718000       2.743901000       0.558171000
C        -2.126398000      -4.983838000      -3.575622000
C        -1.024454000      -5.753926000      -3.241173000
C        -0.097447000      -5.078041000      -2.365626000
C        -0.482998000      -3.783888000      -2.022727000
C         0.177748000      -2.780363000      -1.163950000
C         1.418711000      -3.080749000      -0.535614000
C         2.113546000      -2.166848000       0.297861000
Fe        0.000035000       0.044434000       0.009172000
O        -0.930257000      -0.573095000       1.689841000
O        -1.778173000       0.830867000      -0.487468000
O         0.843770000       0.852127000      -1.635508000
O         0.655153000       1.691382000       0.950459000
```

O	-0.453888000	-1.624560000	-1.031664000
O	1.655995000	-0.951321000	0.556427000
S	-1.162065000	-1.906858000	4.320885000
S	1.649134000	0.978256000	-4.481220000
S	-2.074272000	-3.351406000	-2.810614000
H	3.064548000	4.133871000	-3.125680000
H	3.586819000	3.896608000	-5.687128000
H	2.595033000	1.652361000	-6.710486000
H	2.361157000	3.794166000	-1.039048000
H	1.845968000	-4.057815000	-0.709427000
H	0.816167000	-5.542937000	-2.010154000
H	-0.874292000	-6.765760000	-3.602511000
H	-2.965768000	-5.240286000	-4.208899000
H	-1.980135000	-2.853477000	6.501075000
H	-4.444346000	-1.969253000	6.037886000
H	-4.629231000	-0.703695000	3.745726000
H	-4.192964000	0.239453000	1.772745000
C	1.665576000	3.762448000	1.622146000
C	1.203892000	3.520805000	2.945937000
C	2.399179000	4.953429000	1.358244000
C	1.467266000	4.443631000	3.979796000
C	2.661786000	5.875980000	2.393275000
C	2.197652000	5.625638000	3.707513000
H	0.645774000	2.611807000	3.143777000
H	2.767315000	5.173786000	0.361249000
H	1.107410000	4.243372000	4.986048000
H	3.223145000	6.781104000	2.175180000
H	2.401565000	6.338258000	4.503205000
C	3.426060000	-2.527152000	0.940561000
C	4.052125000	-3.792300000	0.756572000
C	4.065055000	-1.559956000	1.765290000
C	5.284052000	-4.078650000	1.382582000
C	5.296523000	-1.847320000	2.390423000
C	5.911435000	-3.108581000	2.201507000
H	3.596838000	-4.556651000	0.134694000
H	3.585523000	-0.596867000	1.903809000
H	5.748972000	-5.049786000	1.231906000
H	5.771128000	-1.096496000	3.017470000
H	6.860803000	-3.332006000	2.682498000
C	-4.069658000	1.526730000	-0.654689000
C	-3.765390000	2.115084000	-1.913688000
C	-5.399452000	1.623288000	-0.155979000
C	-4.761948000	2.782388000	-2.656270000
C	-6.395513000	2.291347000	-0.899626000
C	-6.081852000	2.873218000	-2.151987000
H	-2.751173000	2.039415000	-2.290892000
H	-5.671125000	1.188219000	0.800635000
H	-4.512290000	3.226129000	-3.616974000
H	-7.406279000	2.356506000	-0.504585000
H	-6.851574000	3.387118000	-2.722953000

$[Fe(dtm)_3]$

67

symmetry c1

Fe	0.012443000	-0.001422000	0.000960000
O	-1.375693000	-0.501070000	-1.370423000
O	-0.186661000	-1.892810000	0.661565000
O	1.479136000	-0.450935000	-1.302631000
O	1.498404000	0.314788000	1.321646000
O	-0.005364000	1.900589000	-0.659842000
O	-1.342798000	0.621811000	1.353984000
C	-0.688076000	2.969202000	-0.273813000
C	-1.618879000	2.962212000	0.799654000
C	-1.908766000	1.801501000	1.566217000
C	-2.042534000	-1.625211000	-1.590727000
C	-1.869586000	-2.806199000	-0.819765000
C	-0.957919000	-2.895876000	0.266302000
C	2.799001000	-0.474736000	-1.182464000
C	3.484668000	-0.157033000	0.020750000
C	2.816458000	0.221057000	1.216333000
H	-2.458577000	-3.677808000	-1.072087000
H	4.565351000	-0.204606000	0.026570000
H	-2.129688000	3.883556000	1.045483000
C	3.587802000	0.538850000	2.434052000
C	4.962057000	0.533760000	2.662907000
C	5.334134000	0.910416000	4.005475000
C	4.249285000	1.206532000	4.814544000
S	2.680466000	1.031374000	3.941828000
H	5.692949000	0.270899000	1.905283000
H	6.362227000	0.957224000	4.348674000
H	4.241049000	1.513232000	5.852418000
C	3.552630000	-0.859668000	-2.391862000
C	4.924135000	-0.982225000	-2.603891000
C	5.276282000	-1.387360000	-3.943538000
C	4.178748000	-1.578007000	-4.767102000
S	2.622309000	-1.260097000	-3.912767000
H	5.666754000	-0.791502000	-1.836117000
H	6.299727000	-1.528874000	-4.274422000
H	4.154890000	-1.879005000	-5.806409000
C	-0.823194000	-4.154398000	1.025594000
C	-1.472475000	-5.377595000	0.873786000
C	-1.061137000	-6.381568000	1.825440000
C	-0.094731000	-5.935857000	2.712350000
S	0.365675000	-4.217943000	2.411798000
H	-2.220948000	-5.565838000	0.111135000
H	-1.467965000	-7.387073000	1.845243000
H	0.385626000	-6.474532000	3.518817000
C	-3.007875000	-1.597882000	-2.707095000
C	-3.855622000	-2.589647000	-3.195606000
C	-4.662341000	-2.171300000	-4.316775000
C	-4.439223000	-0.857461000	-4.695307000

S	-3.196258000	-0.053171000	-3.664932000
H	-3.910050000	-3.588811000	-2.775986000
H	-5.373979000	-2.821080000	-4.815075000
H	-4.902322000	-0.294079000	-5.494966000
C	-2.887693000	1.861282000	2.669398000
C	-3.650771000	2.924883000	3.146230000
C	-4.505417000	2.581442000	4.257269000
C	-4.404327000	1.253664000	4.639315000
S	-3.223944000	0.340991000	3.625841000
H	-3.611466000	3.924085000	2.725077000
H	-5.163027000	3.292458000	4.746090000
H	-4.925537000	0.734917000	5.433400000
C	-0.430618000	4.210284000	-1.030361000
C	-0.969062000	5.487130000	-0.886264000
C	-0.457253000	6.449283000	-1.832368000
C	0.476198000	5.917802000	-2.707250000
S	0.776634000	4.165779000	-2.401340000
H	-1.707370000	5.742600000	-0.133290000
H	-0.771876000	7.487237000	-1.857027000
H	1.013002000	6.410395000	-3.507579000

[Fe(hfaa)$_3$]

43
symmetry c1

Fe	-0.000804000	-0.001938000	-0.003095000
O	-1.638392000	0.112821000	-1.173189000
O	-1.154465000	-1.183555000	1.153864000
O	0.726154000	-1.486317000	-1.156238000
O	1.588531000	-0.406166000	1.172652000
O	0.937204000	1.361949000	-1.158097000
O	-0.465451000	1.587629000	1.146525000
C	1.072019000	2.648550000	-1.040597000
C	0.558553000	3.456304000	-0.003616000
C	-0.184842000	2.850800000	1.031715000
C	-2.822453000	-0.409318000	-1.057455000
C	-3.276911000	-1.238461000	-0.009707000
C	-2.389775000	-1.567565000	1.037266000
C	1.773519000	-2.244683000	-1.032458000
C	2.716127000	-2.210870000	0.016791000
C	2.547890000	-1.274761000	1.059575000
H	-4.288901000	-1.616763000	-0.009567000
H	3.555295000	-2.891401000	0.021643000
H	0.731485000	4.522752000	-0.002621000
C	-3.780934000	-0.047488000	-2.220993000
F	-3.289507000	-0.528384000	-3.445083000
F	-5.065599000	-0.576866000	-2.051642000
F	-3.915706000	1.343201000	-2.350212000
C	-2.872365000	-2.475974000	2.197067000
F	-2.077356000	-3.628007000	2.299657000
F	-2.787895000	-1.807742000	3.428597000

F	-4.198185000	-2.897432000	2.041966000
C	1.940716000	-3.268166000	-2.184724000
F	3.065463000	-4.086315000	-2.026826000
F	0.821364000	-4.110454000	-2.275589000
F	2.066548000	-2.618247000	-3.422222000
C	3.576185000	-1.224102000	2.218844000
F	4.608750000	-2.157435000	2.070175000
F	4.169924000	0.044482000	2.309736000
F	2.957755000	-1.477919000	3.452811000
C	1.894265000	3.295913000	-2.184534000
F	3.197535000	2.774593000	-2.223115000
F	1.310983000	3.037107000	-3.433931000
F	2.005372000	4.685313000	-2.055973000
C	-0.756152000	3.723662000	2.178556000
F	-0.272774000	3.298041000	3.425185000
F	-0.429238000	5.078110000	2.044668000
F	-2.156396000	3.634828000	2.228742000
